# ATAC-seq reveals the roles of chromatin accessibility in the chondrocytes of Kashin–Beck disease compared with primary osteoarthritis

**DOI:** 10.3389/fgene.2023.1169417

**Published:** 2023-05-23

**Authors:** Sen Wang, Yuanji Wang, Xingyu Li, Linlin Yuan, Xiong Guo, Mikko J. Lammi

**Affiliations:** ^1^ School of Public Health, Health Science Center, Xi’an Jiaotong University, Xi’an, Shaanxi, China; ^2^Department of Pharmacy, The First Affiliated Hospital of Northwest University, Xi’an, Shaanxi, China; ^3^Department of Ophthalmology, Shaanxi Eye Hospital, Xi’an People’s Hospital (Xi’an Fourth Hospital), Affiliated Guangren Hospital, School of Medicine, Xi’an Jiaotong University, Xi’an, Shaanxi, China; ^4^ Department of Integrative Medical Biology, University of Umeå, Umeå, Sweden

**Keywords:** Kashin–Beck disease, ATAC-seq, cartilage, chondrocyte, osteoarthritis

## Abstract

**Objective:** This study aimed to investigate the roles of accessible chromatin in understanding the different pathogeneses between Kashin–Beck disease (KBD) and primary osteoarthritis (OA).

**Methods:** Articular cartilages of KBD and OA patients were collected, and after tissue digestion, primary chondrocytes were cultured *in vitro*. Assay for transposase-accessible chromatin with high-throughput sequencing (ATAC-seq) was performed to compare the accessible chromatin differences of chondrocytes between KBD and OA groups. Gene Ontology (GO) and Kyoto Encyclopedia of Genes and Genomes (KEGG) enrichment analyses were executed for the promoter genes. Then, the IntAct online database was used to generate networks of significant genes. Finally, we overlapped the analysis of differentially accessible region (DAR)-associated genes and differentially expressed genes (DEGs) obtained from whole-genomic microarray.

**Results:** We obtained 2,751 total DARs, which contained 1,985 loss and 856 gain DARs and belonged to 11 location distributions. We obtained 218 motifs associated with loss DARs, 71 motifs associated with gain DARs, 30 motif enrichments of loss DARs, and 30 motif enrichments of gain DARs. In total, 1,749 genes are associated with loss DARs, and 826 genes are associated with gain DARs. Among them, 210 promoter genes are associated with loss DARs, and 112 promoter genes are associated with gain DARs. We obtained 15 terms of GO enrichment and 5 terms of KEGG pathway enrichment from loss DAR promoter genes, and 15 terms of GO enrichment and 3 terms of KEGG pathway enrichment from gain DAR promoter genes. We obtained CAPN6 and other 2 overlap genes from loss DARs-vs-down DEGs, AMOTL1 from gain DARs-vs-down DEGs, EBF3 and other 12 overlap genes from loss DARs-vs-up DEGs, and ADARB1 and other 10 overlap genes from 101 gain DARs-vs-up DEGs. These overlap genes were built into 4 gene interaction networks.

**Conclusion:** *FGF7, GPD1L, NFIB, RUNX2,* and *VCAM1* were the overlapped genes from the DAR-associated genes and DEGs. These genes were associated with the abnormal chondrocyte function, which may play crucial roles in different processes between KBD and OA in the way of accessible chromatin.

## Introduction

Kashin–Beck disease (KBD) is one kind of endemic, chronic, and degenerative osteoarthropathy, which is characterized by apoptosis and necrosis of chondrocytes, degradation of cartilage, and extracellular matrix (Guo et al., 2014). Today, it is observed primarily in China, North Korea, and Siberia (Wang et al., 2020). In China, there were still a lot of people living in KBD endemic areas, 98.25 million people were at 379 counties, and 32.5 million people were at 2,047 villages, according to the 2022 Health Statistical Yearbook of China.

KBD is one special type of primary osteoarthritis (OA) with similar clinical manifestations at the late stage. The similar clinical manifestations include morning stiffness, joint pain, enlarged and shortened joints, and dysfunctional and deformed (Wang et al., 2013) joints. According to the diagnostic criteria of KBD of China (WS/T 207-2010 criteria), there are three grades (I–III) in KBD diagnosis. Grade I: thickening of finger joints; grade II: on the basis of grade I, deformity with short fingers (toes); and grade III: on the basis of grade II, deformity with short limb or short stature.

Assay for transposase-accessible chromatin with high-throughput sequencing (ATAC-seq) is a relatively new technique, which is used to define regions of accessible chromatin in cells (Galang et al., 2020). ATAC-seq owns several advantages compared with other sequencing technologies in testing the chromatin of DNase-seq (Merrill et al., 2022). It uses hyperactive Tn5 transposase to cut and ligate adapters simultaneously for high-throughput sequencing at regions of increased accessibility of chromatin. The mapping of inserted ends in genome-wide high-throughput sequencing allows multidimensional analysis of the regulatory landscape of chromatin in a relatively simple protocol for a standard sample of 50,000 cells in a few hours (Buenrostro et al., 2015).

To the best of our knowledge, we have found no other study related to the chromatin accessibility in the chondrocytes of KBD. This is the first time when such features of KBD have been analyzed using the ATAC-seq method. This study was performed to study chromatin accessibility signatures in the chondrocytes of KBD. We propose that changes of accessible chromatin in the chondrocytes may play crucial roles in understanding the different processes between KBD and OA.

## Materials and methods

### Cartilage sample collection

Articular cartilage samples were collected from 11 KBD patients (six females and five males, 55–69 years old, seven grade II and four grade III, respectively) and 11 primary OA patients (six females and five males, 54–67 years old, respectively) matched based on age and sex. The KBD patients from Xi’an city, Yongshou, and Linyou counties in Shaanxi Province of China were diagnosed as grades II and III based on the diagnostic criteria of KBD of China (WS/T 207-2010 criteria). Meanwhile, the primary OA patients came from the non-KBD-endemic areas at Xi’an in Shaanxi Province, according to the Western Ontario and McMaster Universities OA Index (WOMAC). All KBD and OA patients underwent knee arthroplasty or debridement in hospitals. Cartilage samples were stored in liquid nitrogen at −196°C after washing with physiological saline. This investigation was approved by the Human Ethics Committee of Xi’an Jiaotong University.

### *In vitro* culture of chondrocytes

The cartilage samples of the KBD and OA patients were cut and digested with 0.25% trypsin and 0.2% collagenase type II (Sigma, Germany). The cartilage was incubated in trypsin solution overnight at 37°C. Chondrocytes were cultured in a DMEM/F12 culture medium (Gibco, Grand Island, NY), which contained 10% fetal bovine serum (Gibco, Grand Island, NY) in a CO_2_ incubator. Primary cells were used in all experiments.

### ATAC-seq experiment and bioinformatics analysis

The chondrocytes obtained from two KBD patients (one female and one male, 55 and 62 years old, respectively) and two OA patients (one female and one male, 57 and 63 years old, respectively) were used in the ATAC-seq experiment. The protocol followed the one described previously (Buenrostro et al., 2015). The methods included five steps: cell lysis, transposition, amplification, sequencing, and peak calling**.** Briefly, an ice–cold cell lysis buffer was added to the chondrocyte suspension to release nuclei. Tn5 transposed DNA was purified using AMPure DNA magnetic beads and then amplified by PCR. The qualified library was sequenced on an Illumina NovoSeq platform (San Diego, United States) with the PE150 mode. A trimmed read was aligned to the reference genome using Bowtie 2 (Langmead and Salzberg, 2012). All peak calling was performed using MACS-23 (Zhang et al., 2008).

We detected the differentially accessible regions (DARs) between the two sets of samples by comparing the signal values of ATAC-seq data. The DiffBind package in the R program was applied to test potential DARs in consensus peaksets. Significant DARs were considered peaks with a false discovery rate (FDR) < 0.05 and log_2_ fold enrichment >0.5. The ggplot2 package in the R program was utilized to build the volcano plot of DARs. Subsequently, we assessed the clustering of DAR signal values of individual samples between groups. pheatmap was applied to construct the clustering diagram of DARs. We completed the annotation and distribution statistics for the location of DARs in the genome. The ChIPseeker package in the R program was applied to plot the location distribution pie charts of DARs.

Motif scans and enrichment analyses of loss DARs (diminished accessible regions) and gain DARs (enhanced accessible regions) were performed in the JASPAR database using the MEME Suite motifs and kept only when the *p*-value was <0.01 (Heinz et al., 2010).

Gene Ontology (GO) and Kyoto Encyclopedia of Genes and Genomes (KEGG) enrichment analyses were performed for promoter genes. The clusterProfiler package in the R program was applied to build the GO enrichment diagram (*p*-value < 0.05).

### Whole-genomic microarray analysis

Chondrocytes from four KBD (two females and two males, 58–68 years old, respectively) and four primary knee OA (two females and two males, 55–67 years old) patients were divided into four pairs for the microarray analysis. Total RNA was isolated using a minikit (Agilent, CA), following the protocol. Then, 1% agarose gel electrophoresis was performed to check the RNA integrity. Total RNA samples were converted into cDNA using the Amino Allyl MessageAmp aRNA Kit (Ambion, TX). Agilent 44 K human whole-genome oligonucleotide microarray and Feature Extraction 9.3 software were then utilized. A fold change of ≥2.5 or ≤0.4 was considered significant.

### qRT-PCR analysis

In order to validate the microarray results of different experimental groups, we used significant DEGs named DOK5, TRPC6, and EPHA3 as target genes for qRT-PCR. The chondrocytes of five KBD patients (three females and two males, 55–69 years old) and five OA patients (three females and two males, 54–66 years old) were used in the experiments. Total RNA was extracted in the same way with the one in the microarray analysis.

We applied the ABI 7500 RT-PCR system was utilized for the qRT-PCR analysis. We used glyceraldehyde-3-phosphate dehydrogenase (GAPDH) as an endogenous control. The primers and probes NM_018431 [for DOK5], NM_004621 [for TRPC6], and NM_005233 [for EPHA3] were chosen. Comparative analysis between the qRT-PCR data and the microarray data were implemented using paired *t*-tests in SPSS 19.0.

### Transcriptome combined analysis between the ATAC-seq signal and microarray signal

We utilized a transcriptome-combined analysis to elucidate the relationship between the levels of the ATAC-seq signal and gene expression. After dividing the genes of each sample into five classes according to their expression levels, we observed the distribution trends of ATAC-seq signals in the gene body and their upstream and downstream 3-kb regions at different expression levels. Finally, we compared the DAR-associated genes and the DEGs to get the overlapped ones. The DEGs indicated the differential expression genes in chondrocytes between the KBD and OA groups**.**

The IntAct online database (https://www.ebi.ac.uk/intact/home) was used to build the gene networks of the overlap genes mentioned previously. We only selected the direct interaction from the *Homo sapiens* gene.

## Results

### Total peaks and DARs

**FIGURE 1:**
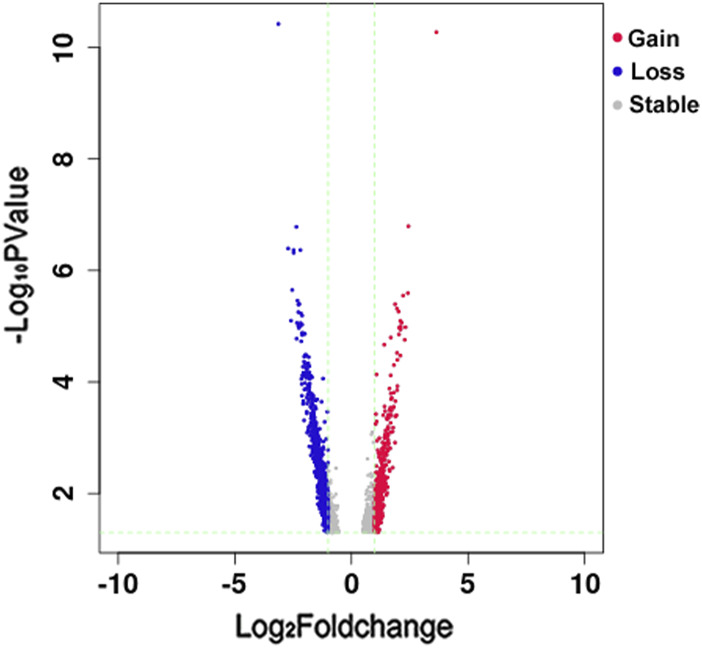
Volcano plot of Loss and Gain DARs. The horizontal axis is the log_2_ fold change of DARs, and the vertical axis is the–log_10_ *p*-value of DARs. Blue represents loss DAR, red represents gain DAR, and gray represents the area with no significant difference. The green trend lines are X = −1 (representing fold change = −2), X = 1 (representing fold change = 2), and Y = 1.3 (representing *p*-value = 0.05).

We obtained 41,193 total peaks in the KBD group and 51,900 in the OA group, of which 37,359 were overlapping peaks between the two groups. By comparing the signal values of the ATAC-seq data between the two sets of samples, we obtained 2,751 total DARs (1,985 loss DARs and 856 gain DARs in the KBD group compared with the OA one). The volcano plot of loss and gain DARs, based on the log_2_ fold change and *p*-value of DARs, is shown in Figure 1.

### Location distribution of DARs

**FIGURE 2:**
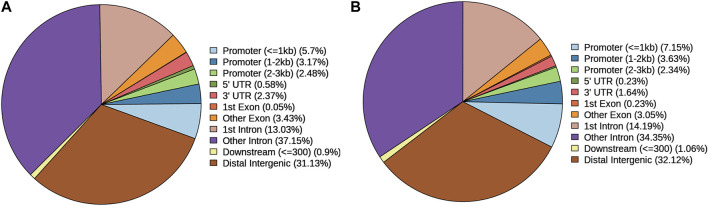
Location distribution percentages of **(A)** loss DARs and **(B)** gain DARs. Icons are the location types in the genome and their proportions of DARs.

The location distribution percentages of gain and loss DARs are shown in Figure 2. The locations included promoters, 5′UTR, 3′UTR, and six other items. No matter whether the loss DAR (Figure 2A) or gain DAR (Figure 2B) was analyzed, the trend of the location ranking was consistent.

### The results of motif enrichment

**FIGURE 3:**
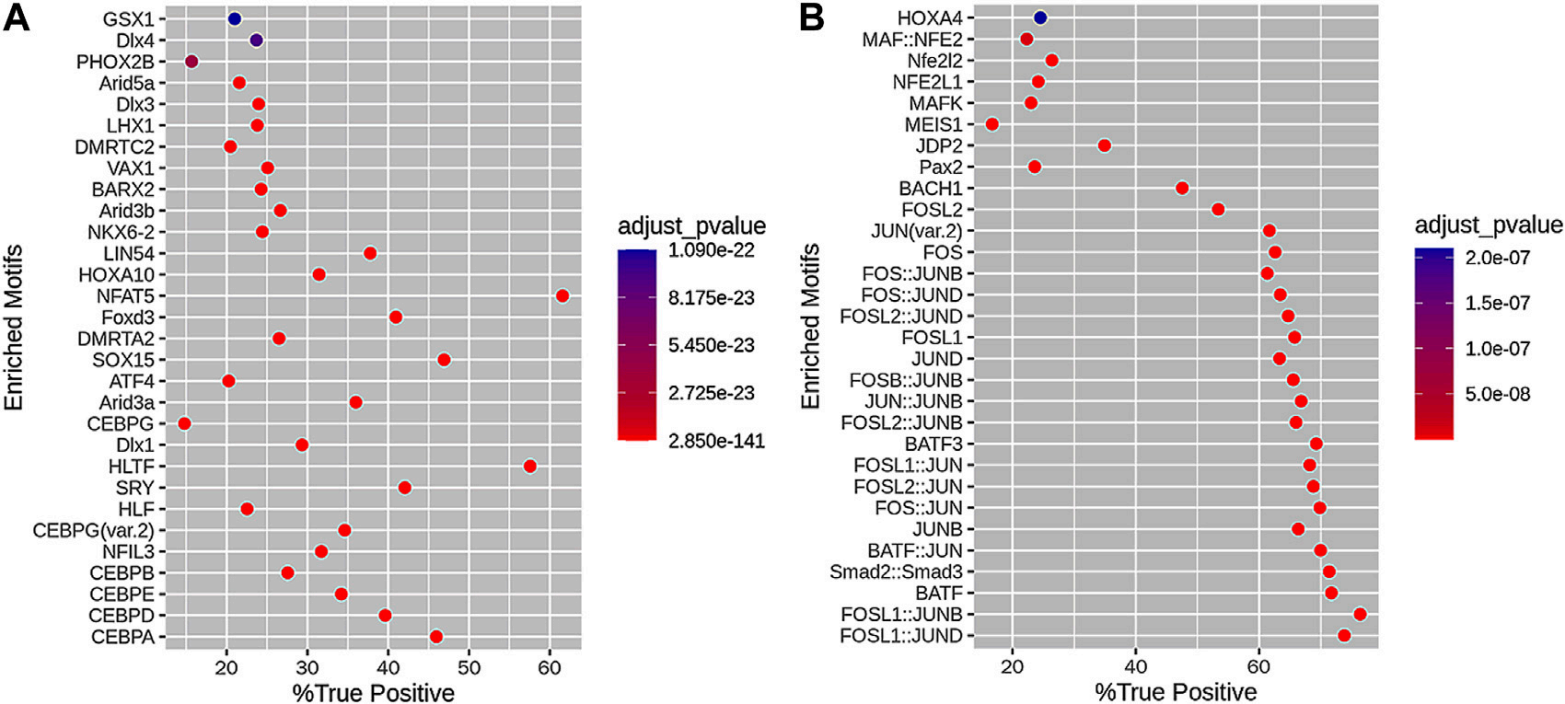
Motif enrichment results of **(A)** loss DARs and **(B)** gain DARs. The horizontal axis is the percentage of DARs with this motif in the DAR set, and the vertical axis is the motif. The color scale indicates the size of the adjusted *p*-value.

We obtained 218 motifs associated with loss DARs and 71 motifs associated with gain DARs. Subsequently, we built 30 motif enrichments of loss DARs and 30 motif enrichments of gain DARs (shown in Figures 3A, B). The motifs are ranked by their adjusted *p*-value.

### Annotation of DAR-associated genes

**FIGURE 4:**
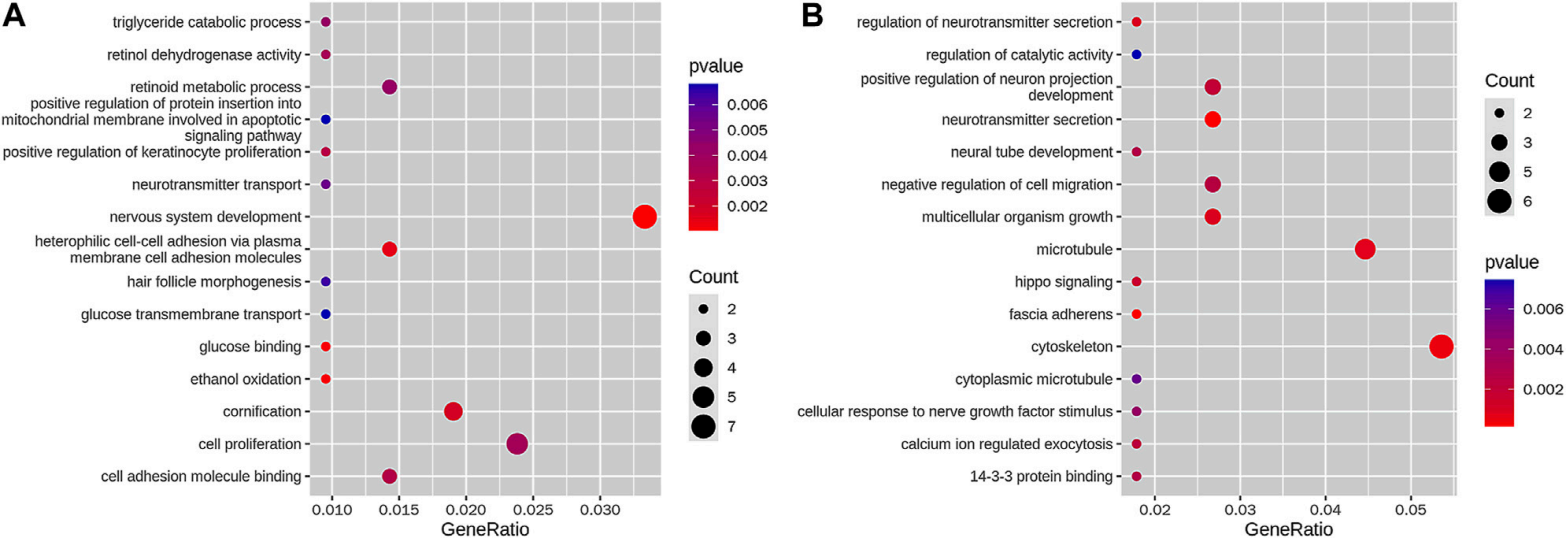
GO enrichment of **(A)** loss DAR promoter genes and **(B)** gain DAR promoter genes. The horizontal axis is a gene ratio, which is the ratio of the gene number in the GO term to the GO term in the gene of the species, and the vertical axis is the GO term; the color in the figure is the size of the *p*-value, and the circle is the size of the number of genes.

Between the KBD and OA groups, 1,749 genes were associated with loss DARs, and 826 genes, with gain DARs. Among them, 210 promoter genes associated with loss DARs and 112 promoter genes associated with gain DARs. We obtained 15 terms of GO enrichment for loss DAR promoter genes and 15 terms of GO enrichment for gain DAR promoter genes (shown in Figures 4A, B). The size and color of the circle represent the number of *p*-values and the gene number in GO.

**FIGURE 5:**
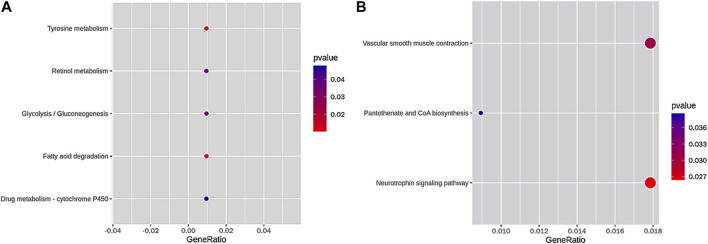
KEGG pathway enrichment of **(A)** loss DAR promoter genes and **(B)** gain DAR promoter genes. The horizontal axis is the gene ratio, which is the ratio of the gene number in the pathway to the pathways in the gene of the species. The vertical axis is the pathway. The color in the figure represents the size of the *p*-value, and the circle indicates the number of genes in the pathway.

Subsequently, we obtained five terms of the KEGG pathway enrichment of loss DAR promoter genes and three terms of the KEGG pathway enrichment of gain DAR promoter genes (shown in Figures 5A, B). The size and color of the circle represent the number of *p*-values and the gene number in the pathway.

### Whole-genomic microarray analysis

The microarray analysis revealed 2,236 upregulated genes and 137 downregulated genes in the chondrocytes of KBD patients compared to the OA patients (shown in Supplementary Table S1).

### qRT-PCR analyses

The genes *DOK5, TRPC6,* and *EPHA6* were selected for validation of the gene expression results in the microarray. It is confirmed that all three genes were upregulated in the KBD chondrocytes, consistent with the microarray data (shown in Supplementary Figure S1).

### Relationship between ATAC-Seq signal intensity and gene expression levels

From the overlap analysis of DAR-associated genes and DEGs, we obtained 3 overlapping genes from 207 loss DARs versus 732 downregulated DEGs (*CAPN6, EVL,* and *PTPN6*); 1 overlapping gene from 111 gain DARs versus 734 downregulated DEGs (*AMOTL1*); 13 overlapping genes from 197 loss DARs versus 5,646 upregulated DEGs (*EBF3, FGF7, FOLH1, GLRA3, JDP2, LGI2, MBNL1, NFIB, NLGN1, RUNX1T1, RUNX2, SPINK6,* and *VCAM1*), and 11 overlapping genes from 101 gain DARs versus 5,648 upregulated DEGs (*ADARB1, BMP2K, CALD1, DYNLT1, GPD1L, IGF2BP2, NRAP, PEG10, RIMS1, SLC22A3,* and *VNN1*).

### Gene interaction networks of the overlap genes

**FIGURE 6:**
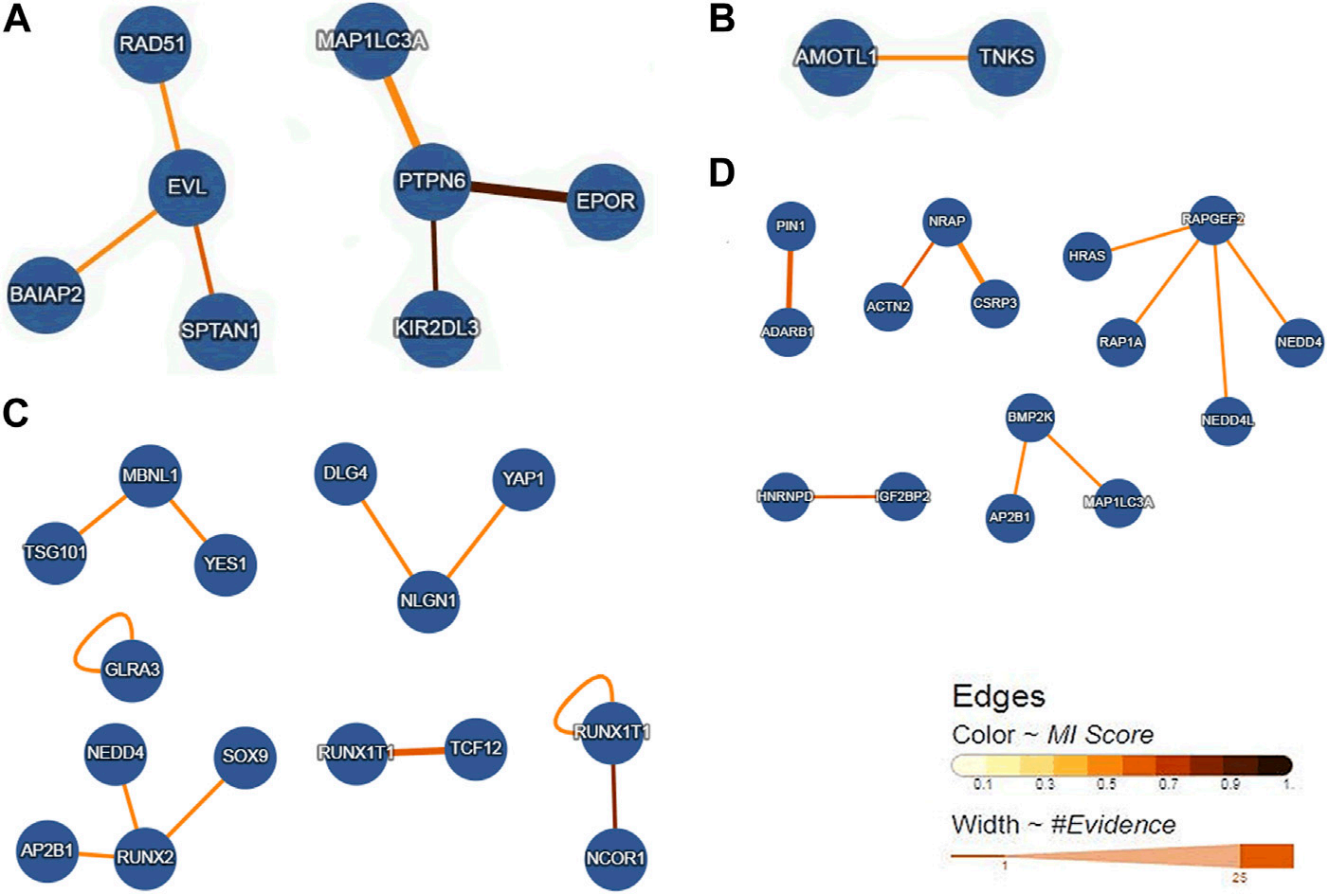
Gene interaction networks of the overlap genes. **(A)** Networks containing the overlap genes from Loss DAR-vs-down DEGs. **(B)** Networks including the overlap genes from gain DAR-vs-down DEGs. **(C)** Networks containing the overlap genes from loss DAR-vs-up DEGs. **(D)** Networks containing the overlap genes from gain DAR-vs-up DEGs.

We obtained four gene interaction networks from each overlapping gene group. The network containing *EVL* and *PTPN6* is shown in Figure 6A, while the network containing *AMOTL1* is shown in Figure 6B. The network containing *MBNL1, NLGN1, RUNX1T1,* and *RUN*X2 is shown in Figure 6C, and *ADARB1, BMP2K, IGF2BP2,* and *NRAP* can be seen in Figure 6D.

## Discussion

This study revealed that *FGF7, GPD1L, NFIB, RUNX2,* and *VCAM1* were overlapping genes between the DAR-associated genes and DEGs. *FGF7, NFIB, RUNX2,* and *VCAM1* belonged to loss DAR and upregulated DEGs. *GPD1L* belonged to gain DARs and upregulated DEGs. These significant genes are associated with the abnormal chondrocyte function, which may play crucial roles in KBD in the way of accessible chromatin.

It has been shown that growth plate chondrocytes express the fibroblast growth factor 7 (FGF7) (Lazarus et al., 2007). FGF7 is also expressed significantly higher in OA meniscal cells than the normal meniscal cells (Sun et al., 2010). In this study, the relation of the FGF7 in comparison with loss DARs and upregulated DEGs was observed, indicating that its abnormal expression in the KBD is more serious than that in OA cells. The diminished accessible region may cause upregulation.

Articular cartilage is a tissue under a low oxygen tension, and the chondrocytes are adapted to the hypoxic condition. Specific hypoxia-inducible factors (HIFs) are involved in chondrogenesis, regulated by transcription factors such as SOX9 (Zhang et al., 2015). In this role, HIF-1α is an important regulator which contributes to the synthesis of the chondrocytes’ extracellular matrix (Zhai et al., 2017). The gene *GPD1L* has been implicated in circ-0001946/miR-21/GPD1L/HIF-1α axis. *GPD1L* is a main regulator for the hydroxylation of HIF-1α, which can lead to the decrease of HIF-1α expression (Kelly et al., 2011). HIF-1α can protect the articular cartilage in the way of promoting the chondrocyte phenotype, supporting the adaptation to a hypoxic environment and maintaining the chondrocyte viability (Zhang et al., 2015). Although primary chondrocytes can resist heat shock protein (Hsp)-associated stress (Kaarniranta et al., 2001), HIF-1α-induced HSP70 overexpression can increase the expression of ECM genes and the cell viability and protect chondrocytes from apoptosis. It may indicate that *GPD1L* participates in the modulation of chondrocyte apoptosis (Tsuchida et al., 2014). Meanwhile, *GPD1L*-knockdown chondrocytes presented a higher apoptotic rate (Zhai et al., 2017). *GPD1L* was involved in gain DARs and upregulated DEGs. It appears to indicate that the damage to the KBD chondrocytes is more serious than the OA chondrocytes.

This study revealed that *NFIB* was involved in loss DARs and upregulated DEGs. Consistent with the transient activation of the *NFIB* gene in chondrogenesis, dominant-negative mutations in *NFIB* can interfere with chondrogenesis (Uchihashi et al., 2007). The overexpression of *NFIB* increased SOX9 and COL2A1 expression (Nagy et al., 2011). Previous findings indicated that the over-expression of *NFIB* can increase the expression of genes related to the synthesis of ECM and proliferation of chondrocytes. However, the over-expression of *NFIB* can decrease the expression of genes related to the degradation of ECM and differentiation of chondrocytes (Pan et al., 2021). *NFIB* was associated with loss DARs and upregulated DEGs. It may indicate the abnormal chondrocyte function of KBD is more serious than that of OA.

It is well known that *RUNX2* can regulate the proliferation and differentiation of chondrocytes that promote endochondral ossification of chondrocytes to a hypertrophic-like state (Zhang et al., 2022). It is an important transcription factor for the maturation of chondrocytes, which induces the expression of collagen X in the maturation process (Zhang et al., 2019). *RUNX2* was involved in loss DARs and upregulated DEGs. This suggests a stronger involvement of *RUNX* in regulating the chondrocyte function in KBD and OA.

*VCAM1* is a known pro-inflammatory gene (Harasymowicz et al., 2021). Its influence for chondrocytes, which was tested by flow cytometry, includes inflammation and metabolic alterations (Ragni et al., 2020). In addition, *VCAM1* revealed the relation of loss DARs and upregulation of DEGs. It is interesting that the repression of *VCAM1* limited the inflammation and apoptosis in the murine OA model related to the PI3K/Akt pathway (Jiang et al., 2023).

In summary, a number of associations between DARs of chromatin and DEGs were revealed in this study. They may provide a new insight into understanding the different processes between KBD and OA. Further study is warranted to identify the roles of the finding in this study.

## Data Availability

The original contributions presented in the study are publicly available. This data can be found here: https://www.ncbi.nlm.nih.gov/bioproject/PRJNA953668.
