# Supplementary material for: ATAC-seq reveals the roles of chromatin accessibility in the chondrocytes of Kashin–Beck disease compared with primary osteoarthritis
Source: Front Genet. 2023 May 23;14:1169417. doi: 10.3389/fgene.2023.1169417 (PMC10241996; doi:10.3389/fgene.2023.1169417)
Supplement: Supplementary file 1 [file DataSheet1.DOCX]

# Supplementary Figure and Table





Figure S1. Bars showing the expression values of selected genes measured by microarray (blue bars, n=4) and qRT-PCR (red bars, n=5). Values represent Mean±SD. **P*<0.01, ^#^*P*<0.05.

Table S1 Differential expressed gene in chondrocytes between KBD and OA

| Gene symbol | Accession number | Fold change |
| --- | --- | --- |
| DDX3Y | NM_004660 | 207.9737 |
| RPS4Y1 | NM_001008 | 97.09006 |
| HSPB3 | NM_006308 | 80.70778 |
| CYorf14 | NR_001544 | 80.2229 |
| NAV3 | NM_014903 | 73.62779 |
| TOX | NM_014729 | 47.08359 |
| HERPUD2 | BC049371 | 47.00277 |
| GREM2 | NM_022469 | 46.01588 |
| EGR2 | NM_000399 | 43.02517 |
| COL13A1 | NM_005203 | 41.51779 |
| ASPM | NM_018136 | 40.04147 |
| PLAT | NM_000930 | 39.25624 |
| ABCA13 | NM_152701 | 38.56484 |
| EPHA3 | NM_005233 | 37.58152 |
| APXL | NM_001649 | 37.13236 |
| DKK1 | NM_012242 | 36.43343 |
| TNFRSF19 | NM_148957 | 36.1378 |
| SPBC25 | NM_020675 | 35.85088 |
| FRMD4A | NM_018027 | 35.07647 |
| DCAMKL1 | NM_004734 | 32.4884 |
| RNF150 | NM_020724 | 32.2143 |
| SAMD9 | NM_017654 | 30.34126 |
| FGF5 | NM_004464 | 29.89898 |
| SYNPO2 | AL833294 | 29.38645 |
| FLRT2 | NM_013231 | 29.35677 |
| FLRT3 | NM_198391 | 29.30738 |
| MEOX2 | NM_005924 | 28.77873 |
| RPS4Y2 | NM_001039567 | 28.67429 |
| SGCG | NM_000231 | 28.05354 |
| SPINK5L3 | AK001520 | 26.54094 |
| BEX1 | NM_018476 | 25.29412 |
| DYX1C1 | NM_130810 | 24.17548 |
| GPR1 | NM_005279 | 22.94622 |
| CXCL12 | NM_000609 | 21.46942 |
| EIF1AY | NM_004681 | 21.40345 |
| HTR7 | NM_019859 | 21.03927 |
| GREM1 | NM_013372 | 20.59732 |
| KCNK2 | NM_001017424 | 20.13085 |
| SLC38A4 | NM_018018 | 20.06427 |
| C20orf39 | NM_024893 | 20.00851 |
| NME5 | NM_003551 | 19.79658 |
| POSTN | NM_006475 | 19.78583 |
| PTN | NM_002825 | 19.59396 |
| ITGA6 | NM_000210 | 19.4924 |
| RGS4 | NM_005613 | 19.38853 |
| UBQLN1 | NM_013438 | 19.38241 |
| ADH1A | NM_000667 | 19.21238 |
| C8orf48 | BC031245 | 18.97637 |
| ARHGAP18 | NM_033515 | 18.88869 |
| SHANK2 | NM_012309 | 18.57463 |
| SALF | NM_172311 | 18.42899 |
| IL7 | NM_000880 | 18.40156 |
| FAT4 | AK026709 | 18.39826 |
| PSAT1 | NM_058179 | 18.36703 |
| IL7R | NM_002185 | 18.22817 |
| APCDD1 | NM_153000 | 17.91681 |
| TTC30B | NM_152517 | 17.90787 |
| FMN2 | NM_020066 | 17.87081 |
| FAM13C1 | NM_001001971 | 17.69252 |
| PITX2 | NM_153426 | 17.62045 |
| TEK | NM_000459 | 17.59139 |
| MMP19 | NM_002429 | 17.43244 |
| FRAS1 | NM_025074 | 17.38296 |
| ZFY | NM_003411 | 17.30609 |
| MAGI2 | NM_012301 | 17.19993 |
| OCLN | NM_002538 | 17.17343 |
| ZWINT | NM_001005414 | 17.14819 |
| DOK6 | NM_152721 | 17.06979 |
| CDH2 | NM_001792 | 16.99223 |
| LIN7A | NM_004664 | 16.85669 |
| SULF1 | NM_015170 | 16.62695 |
| ATP10A | NM_024490 | 16.57272 |
| PRMT6 | NM_018137 | 16.52086 |
| CXorf10 | AK021694 | 16.30284 |
| SEMA6D | NM_153618 | 16.25686 |
| LPPR4 | NM_014839 | 15.93457 |
| PLK4 | NM_014264 | 15.42314 |
| ALPK2 | NM_052947 | 15.42127 |
| CX3CR1 | NM_001337 | 15.37443 |
| C6orf188 | NM_153711 | 15.27696 |
| VGLL3 | NM_016206 | 15.19203 |
| CYorf15A | NM_001005852 | 15.09083 |
| FRG1 | AK127309 | 15.0671 |
| STMN2 | NM_007029 | 14.95925 |
| TFPI2 | NM_006528 | 14.94372 |
| C11orf41 | U10991 | 14.66576 |
| LEP | NM_000230 | 14.59039 |
| RHBDL2 | NM_017821 | 14.59006 |
| DOCK10 | NM_014689 | 14.5024 |
| CCDC102B | NM_024781 | 14.35374 |
| LQK1 | AY030238 | 14.24046 |
| C18orf24 | NM_145060 | 14.09036 |
| KRTAP1-5 | NM_031957 | 13.92098 |
| MYH1 | NM_005963 | 13.90274 |
| FLJ21986 | NM_024913 | 13.85099 |
| ACTA2 | NM_001613 | 13.83576 |
| SLITRK6 | NM_032229 | 13.79222 |
| PTGIS | NM_000961 | 13.78285 |
| MMP7 | NM_002423 | 13.75925 |
| TTTY15 | NR_001545 | 13.69717 |
| F2R | NM_001992 | 13.64311 |
| NEK10 | NM_001031741 | 13.60874 |
| PLCB1 | NM_182734 | 13.60267 |
| C1orf24 | NM_052966 | 13.58538 |
| RGS20 | NM_170587 | 13.47219 |
| FNDC6 | NM_144717 | 13.39393 |
| PCDH9 | NM_020403 | 13.34552 |
| NIPSNAP3A | NM_015469 | 13.34074 |
| APOB | NM_000384 | 13.28225 |
| ZIC1 | NM_003412 | 13.19033 |
| SAMD9L | NM_152703 | 13.12855 |
| SPTLC2L | AK075271 | 12.99126 |
| ARMC4 | NM_018076 | 12.97822 |
| ACOX2 | NM_003500 | 12.97288 |
| BCKDHB | NM_000056 | 12.92385 |
| C2orf38 | NM_198557 | 12.91465 |
| ANK1 | NM_000037 | 12.91275 |
| MYL1 | NM_079422 | 12.89638 |
| TRPC6 | NM_004621 | 12.57312 |
| LRRC16 | NM_017640 | 12.57 |
| STMN1 | NM_203401 | 12.56177 |
| NRXN3 | NM_004796 | 12.55208 |
| NMU | NM_006681 | 12.54851 |
| HCAP-G | NM_022346 | 12.51578 |
| ANGPTL7 | NM_021146 | 12.4534 |
| KRT34 | NM_021013 | 12.27837 |
| SLC1A3 | NM_004172 | 12.19927 |
| FLJ34870 | NM_207481 | 12.18085 |
| RAB12 | NM_001025300 | 12.069 |
| MGC10993 | NM_030577 | 12.03876 |
| KIAA1345 | AB037766 | 11.94139 |
| CENPK | NM_022145 | 11.93427 |
| KIF20A | NM_005733 | 11.91127 |
| TTC30A | NM_152275 | 11.72503 |
| MGC33648 | NM_153706 | 11.68468 |
| ASPA | NM_000049 | 11.67234 |
| USP9Y | NM_004654 | 11.63063 |
| ACTB | NM_001101 | 11.57434 |
| FLJ43339 | NM_207380 | 11.48722 |
| DSCR1L1 | NM_005822 | 11.41517 |
| WDR5B | NM_019069 | 11.36602 |
| CDCA7 | NM_031942 | 11.28111 |
| NLGN4Y | NM_014893 | 11.21043 |
| DSCR1 | NM_004414 | 11.20499 |
| TCBA1 | NM_001040214 | 11.1889 |
| RAD51 | NM_002875 | 11.03752 |
| LOXL1 | NM_005576 | 10.98983 |
| CH25H | NM_003956 | 10.97717 |
| KIAA1729 | NM_053042 | 10.79357 |
| SOHLH2 | NM_017826 | 10.74192 |
| PRKY | NM_002760 | 10.73674 |
| HECW2 | NM_020760 | 10.71255 |
| C18orf4 | NM_032160 | 10.53769 |
| LMO7 | NM_005358 | 10.53372 |
| PTX3 | NM_002852 | 10.40642 |
| KIAA1189 | NM_020711 | 10.36068 |
| MKX | NM_173576 | 10.3209 |
| MCM10 | NM_182751 | 10.29145 |
| SORBS2 | NM_021069 | 10.27599 |
| GDF10 | NM_004962 | 10.16151 |
| SPINK2 | NM_021114 | 10.16002 |
| STARD13 | NM_178006 | 10.05153 |
| HEY1 | NM_012258 | 10.00469 |
| C5orf13 | NM_004772 | 10.00329 |
| KCNMA1 | NM_001014797 | 9.960185 |
| HEY2 | NM_012259 | 9.937505 |
| CDCA1 | NM_145697 | 9.901129 |
| COL21A1 | NM_030820 | 9.893338 |
| PRSS35 | NM_153362 | 9.861334 |
| ZDHHC21 | NM_178566 | 9.823559 |
| STAU2 | AK002152 | 9.686074 |
| ZNF135 | NM_003436 | 9.683692 |
| SHC4 | NM_203349 | 9.676918 |
| C8orf16 | NM_001014439 | 9.656446 |
| KLF6 | NM_001008490 | 9.647539 |
| ITGA4 | NM_000885 | 9.610618 |
| FLJ20105 | NM_001009954 | 9.590607 |
| CENPA | NM_001809 | 9.534761 |
| KIAA0746 | NM_015187 | 9.517496 |
| W60781 | W60781 | 9.450754 |
| ST8SIA1 | NM_003034 | 9.439202 |
| KCNB1 | NM_004975 | 9.380205 |
| KIAA1217 | NM_019590 | 9.362191 |
| PSPH | NM_004577 | 9.340637 |
| SHOX | U89331 | 9.336282 |
| ITGA8 | NM_003638 | 9.329716 |
| ZNF471 | AB037817 | 9.310651 |
| ADH6 | BX647987 | 9.297021 |
| ABCA8 | NM_007168 | 9.259551 |
| RIMS1 | NM_014989 | 9.194739 |
| LCMT2 | NM_014793 | 9.157915 |
| NEDD9 | NM_006403 | 9.123943 |
| C6orf117 | NM_138409 | 9.104686 |
| FLJ30901 | AK055463 | 9.10214 |
| RTN1 | NM_021136 | 9.100165 |
| PSCA | NM_005672 | 9.064873 |
| HNT | NM_016522 | 8.988546 |
| PLCB4 | NM_000933 | 8.98716 |
| RAPH1 | NM_025252 | 8.979425 |
| USP34 | AL050376 | 8.952656 |
| U85992 | U85992 | 8.940555 |
| DKFZp586C0721 | AL137734 | 8.910049 |
| HAS2 | NM_005328 | 8.904441 |
| KNTC2 | NM_006101 | 8.893986 |
| ZNF143 | U09850 | 8.882396 |
| AQP1 | NM_198098 | 8.87046 |
| MDM1 | NM_020128 | 8.850533 |
| ZNF300 | NM_052860 | 8.835151 |
| ASB5 | NM_080874 | 8.808236 |
| CD24 | L33930 | 8.759546 |
| TP53I3 | NM_004881 | 8.737807 |
| TLR4 | NM_138554 | 8.718678 |
| DLG7 | NM_014750 | 8.701157 |
| CCNL2 | NM_001039577 | 8.696157 |
| NPNT | NM_001033047 | 8.675315 |
| LHPP | NM_022126 | 8.617506 |
| FLNB | AK022486 | 8.598371 |
| FLJ31659 | NM_153027 | 8.581401 |
| CSTF2T | NM_015235 | 8.526829 |
| C3orf14 | NM_020685 | 8.490092 |
| CCDC13 | AK058196 | 8.479265 |
| SLIT2 | NM_004787 | 8.468259 |
| FLJ13646 | NM_024584 | 8.460782 |
| KLF2 | NM_016270 | 8.459159 |
| RNF128 | NM_194463 | 8.447371 |
| CEP55 | NM_018131 | 8.413515 |
| HOMER2 | NM_199330 | 8.407352 |
| CDCP1 | NM_022842 | 8.363647 |
| ADAM23 | NM_003812 | 8.348285 |
| MYO10 | BC108736 | 8.344254 |
| LEF1 | NM_016269 | 8.33839 |
| SPARCL1 | NM_004684 | 8.277623 |
| SCN9A | NM_002977 | 8.268171 |
| CRTAM | NM_019604 | 8.253968 |
| TFDP1 | NM_007111 | 8.234868 |
| PHF5A | NM_032758 | 8.234217 |
| MPP4 | NM_033066 | 8.207021 |
| TPM1 | NM_001018004 | 8.196409 |
| IFI27 | NM_005532 | 8.188032 |
| HTR2A | NM_000621 | 8.181563 |
| TRIM7 | NM_033342 | 8.158544 |
| MAGI3 | NM_020965 | 8.15162 |
| SVEP1 | AK027870 | 8.123701 |
| IGHA1 | AK128476 | 8.094649 |
| IFT80 | NM_020800 | 8.064108 |
| C10orf114 | NM_001010911 | 8.040789 |
| ESCO2 | NM_001017420 | 8.030056 |
| MYOZ2 | NM_016599 | 8.019841 |
| GPR124 | NM_032777 | 7.984943 |
| MGC16385 | NM_145039 | 7.968489 |
| KIAA1407 | NM_020817 | 7.964464 |
| IGFBP5 | NM_000599 | 7.932666 |
| ANXA1 | NM_000700 | 7.929334 |
| HIST1H4A | NM_003538 | 7.925802 |
| FANCA | NM_000135 | 7.903212 |
| L3MBTL2 | AK057177 | 7.898345 |
| C8orf34 | NM_052958 | 7.878017 |
| C6orf142 | NM_138569 | 7.855493 |
| BCL2A1 | NM_004049 | 7.84385 |
| KALRN | NM_001024660 | 7.84156 |
| LXN | NM_020169 | 7.840181 |
| CYP4V2 | NM_207352 | 7.823976 |
| C8orf47 | NM_173549 | 7.817542 |
| THBS1 | NM_003246 | 7.799316 |
| HBB | NM_000518 | 7.77724 |
| ZNF323 | NM_030899 | 7.763717 |
| SEPP1 | NM_005410 | 7.741866 |
| C7orf29 | NM_138434 | 7.737744 |
| RGS5 | NM_003617 | 7.707761 |
| C1orf113 | NM_024676 | 7.707386 |
| CASC5 | NM_170589 | 7.694816 |
| EXO1 | NM_003686 | 7.688399 |
| GPR30 | NM_001505 | 7.688283 |
| PRKCDBP | NM_145040 | 7.67441 |
| EMX2 | NM_004098 | 7.664325 |
| IGFBP3 | NM_001013398 | 7.651074 |
| AADAT | NM_016228 | 7.64949 |
| ZFP2 | NM_030613 | 7.647928 |
| KIAA0101 | NM_014736 | 7.642498 |
| ZNF642 | NM_198494 | 7.641734 |
| WDR78 | NM_024763 | 7.640495 |
| EMX2OS | NR_002791 | 7.629466 |
| LRRN6A | NM_032808 | 7.627336 |
| VPS13A | NM_033305 | 7.617783 |
| PRKG1 | NM_006258 | 7.616164 |
| PLEKHG2 | NM_022835 | 7.608499 |
| CDKN3 | NM_005192 | 7.607764 |
| FSIP1 | NM_152597 | 7.596166 |
| MGC39715 | NM_152628 | 7.584969 |
| NEK1 | NM_012224 | 7.530908 |
| CD248 | NM_020404 | 7.529823 |
| DSTN | NM_001011546 | 7.510339 |
| ALS2CR19 | NM_205863 | 7.500673 |
| SPA17 | NM_017425 | 7.48768 |
| SACS | NM_014363 | 7.472777 |
| BRCA1 | NM_007295 | 7.45871 |
| E2F2 | NM_004091 | 7.441853 |
| TM4SF4 | NM_004617 | 7.440534 |
| RSU1 | NM_012425 | 7.418282 |
| FBXL17 | BC018548 | 7.400049 |
| FCRL4 | NM_031282 | 7.395668 |
| DENND4A | AL833317 | 7.377519 |
| RBMS3 | NM_001003793 | 7.375394 |
| ZNF70 | NM_021916 | 7.372054 |
| CLEC3A | NM_005752 | 7.370018 |
| DHFR | NM_000791 | 7.367358 |
| CDS2 | NM_003818 | 7.363075 |
| MARVELD1 | NM_031484 | 7.362693 |
| SLC4A4 | NM_003759 | 7.355945 |
| ZNF334 | NM_199441 | 7.34401 |
| DOK5 | NM_018431 | 7.310646 |
| IRX3 | NM_024336 | 7.309347 |
| ZNF469 | AB058761 | 7.281617 |
| DMXL1 | NM_005509 | 7.275449 |
| FBN2 | NM_001999 | 7.251472 |
| CXorf15 | NM_018360 | 7.243063 |
| WFDC13 | NM_172005 | 7.23463 |
| CDC45L | NM_003504 | 7.233071 |
| GDF6 | NM_001001557 | 7.225005 |
| SYNC1 | NM_030786 | 7.203532 |
| DAPK1 | NM_004938 | 7.197698 |
| ANGPT1 | NM_001146 | 7.178418 |
| PLEKHG4 | NM_015432 | 7.17814 |
| MGC52057 | NM_194317 | 7.164765 |
| TMOD2 | NM_014548 | 7.145564 |
| MEST | NM_002402 | 7.144274 |
| CORT | NM_001302 | 7.14362 |
| UHRF1 | NM_013282 | 7.113564 |
| NEXN | NM_144573 | 7.109985 |
| EBI2 | NM_004951 | 7.096936 |
| MRVI1 | NM_130385 | 7.067371 |
| KIAA1377 | NM_020802 | 7.036389 |
| DOCK8 | NM_203447 | 7.006412 |
| HOXD1 | NM_024501 | 7.003552 |
| C9orf93 | NM_173550 | 7.002948 |
| ADH1C | NM_000669 | 6.987641 |
| PPAPDC2 | NM_203453 | 6.977528 |
| CAV2 | NM_001233 | 6.977074 |
| FLJ33996 | AK091315 | 6.974453 |
| PRDM12 | NM_021619 | 6.973005 |
| CDGAP | NM_020754 | 6.949357 |
| C12orf48 | NM_017915 | 6.895775 |
| STXBP5 | NM_139244 | 6.876681 |
| GALNT13 | NM_052917 | 6.863913 |
| ADRA1B | NM_000679 | 6.858543 |
| SH3KBP1 | NM_031892 | 6.83327 |
| VPS13B | NM_017890 | 6.82903 |
| TNFRSF11B | NM_002546 | 6.821591 |
| HIPK2 | NM_022740 | 6.81445 |
| TAF5L | NM_014409 | 6.787005 |
| ZNF239 | NM_005674 | 6.781545 |
| NBEA | NM_015678 | 6.779046 |
| HEYL | NM_014571 | 6.772818 |
| F13A1 | NM_000129 | 6.771313 |
| B3GALNT1 | NM_033169 | 6.766732 |
| APITD1 | NM_199295 | 6.760138 |
| HDAC9 | NM_014707 | 6.754537 |
| SLC16A6 | NM_004694 | 6.730934 |
| EDIL3 | NM_005711 | 6.730229 |
| ZFHX4 | NM_024721 | 6.715942 |
| MKL2 | NM_014048 | 6.714952 |
| TTC28 | AK092338 | 6.70726 |
| DZIP3 | NM_014648 | 6.705441 |
| DTL | NM_016448 | 6.69539 |
| ANKRD29 | NM_173505 | 6.693862 |
| TSGA14 | NM_018718 | 6.692838 |
| CRISPLD1 | NM_031461 | 6.687815 |
| EGFL6 | NM_015507 | 6.66744 |
| ZNF588 | NM_016220 | 6.615262 |
| MXRA5 | NM_015419 | 6.610971 |
| STXBP6 | NM_014178 | 6.585763 |
| APLN | NM_017413 | 6.576275 |
| FLJ35946 | AK093265 | 6.55618 |
| CALD1 | NM_033138 | 6.544713 |
| DTWD2 | NM_173666 | 6.505613 |
| FLJ30064 | NM_001039906 | 6.50545 |
| SPATA17 | NM_138796 | 6.498757 |
| MAWBP | NM_022129 | 6.489339 |
| CYR61 | NM_001554 | 6.455747 |
| ELN | BC065566 | 6.449829 |
| RPESP | NM_153225 | 6.439673 |
| KRTAP1-1 | NM_030967 | 6.437227 |
| KIAA1913 | NM_052913 | 6.433995 |
| TTC26 | NM_024926 | 6.389698 |
| LFNG | NM_001040167 | 6.377026 |
| ZBTB26 | NM_020924 | 6.366028 |
| ANLN | NM_018685 | 6.360009 |
| DKFZp313P036 | BX537874 | 6.356481 |
| C14orf56 | AK024445 | 6.354262 |
| STEAP4 | NM_024636 | 6.351884 |
| SHCBP1 | NM_024745 | 6.339824 |
| S100A10 | NM_002966 | 6.334371 |
| PCDH18 | NM_019035 | 6.320802 |
| FLJ20366 | NM_017786 | 6.318837 |
| FLJ34306 | NM_199340 | 6.274307 |
| MYH11 | NM_022844 | 6.272759 |
| GLIPR1 | NM_006851 | 6.268737 |
| EGR1 | NM_001964 | 6.260473 |
| IRF8 | NM_002163 | 6.260071 |
| WDR52 | AK002004 | 6.258537 |
| GALNTL4 | AF131852 | 6.249636 |
| FLJ37549 | NM_152605 | 6.246173 |
| MRCL3 | NM_006471 | 6.228893 |
| SERPINE1 | NM_000602 | 6.227212 |
| CSTA | NM_005213 | 6.221947 |
| SLC7A11 | NM_014331 | 6.220827 |
| DHFRL1 | NM_176815 | 6.203675 |
| YWHAB | NM_003404 | 6.202894 |
| GALNT3 | NM_004482 | 6.190929 |
| RNF170 | NM_030954 | 6.188685 |
| ALS2CR8 | NM_024744 | 6.169373 |
| TMEM16D | NM_178826 | 6.147665 |
| PTGDS | NM_000954 | 6.141037 |
| IGF2BP3 | NM_006547 | 6.124996 |
| KIAA1009 | NM_014895 | 6.124787 |
| KCTD16 | NM_020768 | 6.115897 |
| MSRA | NM_012331 | 6.092089 |
| C8orf15 | NM_001033662 | 6.085567 |
| ADAMTS6 | NM_197941 | 6.076538 |
| FLJ46385 | NM_001001675 | 6.071377 |
| CNN1 | NM_001299 | 6.071191 |
| LRRCC1 | NM_033402 | 6.069522 |
| PNMA2 | NM_007257 | 6.051113 |
| C21orf122 | BC004343 | 6.036259 |
| MYLK | NM_053025 | 6.027019 |
| TIGD6 | NM_030953 | 6.01723 |
| PUS7L | NM_031292 | 6.009709 |
| JRK | NM_003724 | 6.009572 |
| SPOCD1 | AK097227 | 6.007836 |
| COL14A1 | NM_021110 | 6.005309 |
| PTGER3 | NM_000957 | 5.989592 |
| STYK1 | NM_018423 | 5.98365 |
| NICN1 | NM_032316 | 5.981756 |
| CITED2 | NM_006079 | 5.970307 |
| HELLS | NM_018063 | 5.962479 |
| SCRN3 | NM_024583 | 5.958754 |
| PRICKLE1 | NM_153026 | 5.957427 |
| PSG8 | NM_182707 | 5.957037 |
| EGR3 | NM_004430 | 5.949026 |
| AP4S1 | NM_007077 | 5.925583 |
| ALDH4A1 | NM_170726 | 5.92494 |
| BUB1B | NM_001211 | 5.91964 |
| WISP1 | NM_003882 | 5.917349 |
| TBC1D8B | NM_017752 | 5.916126 |
| FLJ41747 | AK123741 | 5.914146 |
| NR2F1 | NM_005654 | 5.911677 |
| EYA2 | NM_172113 | 5.909591 |
| ZAK | NM_016653 | 5.903654 |
| GINS2 | NM_016095 | 5.89296 |
| IRAK1BP1 | NM_001010844 | 5.888887 |
| ZNF189 | NM_197977 | 5.884745 |
| MOCOS | NM_017947 | 5.880801 |
| DKK4 | NM_014420 | 5.876075 |
| CDC25C | NM_001790 | 5.873678 |
| ABLIM3 | NM_014945 | 5.862858 |
| FBLIM1 | NM_017556 | 5.861104 |
| SUOX | NM_000456 | 5.859103 |
| NQO1 | NM_000903 | 5.852162 |
| GALNT12 | NM_024642 | 5.833878 |
| CENPF | NM_016343 | 5.830508 |
| C8orf13 | NM_053279 | 5.827031 |
| ADAMTSL3 | NM_207517 | 5.819188 |
| RRM2 | NM_001034 | 5.811092 |
| KIF15 | NM_020242 | 5.806085 |
| TSPAN2 | NM_005725 | 5.759146 |
| LPL | NM_000237 | 5.75444 |
| ANXA2 | NM_001002857 | 5.752913 |
| POLQ | NM_199420 | 5.743705 |
| PCDHA11 | NM_018902 | 5.739966 |
| DNAJC19 | NM_145261 | 5.73002 |
| RAC2 | NM_002872 | 5.710161 |
| C2orf13 | NM_173545 | 5.708511 |
| CDC7 | NM_003503 | 5.706375 |
| EFHC2 | NM_025184 | 5.706329 |
| PIGN | NM_176787 | 5.701817 |
| SCN8A | NM_014191 | 5.698492 |
| PDE4DIP | AB042555 | 5.686089 |
| KIAA1383 | BC036663 | 5.683721 |
| MAP1B | NM_005909 | 5.680787 |
| CCDC53 | NM_016053 | 5.675933 |
| PCBD2 | NM_032151 | 5.675872 |
| MGC16169 | NM_033115 | 5.675568 |
| GLYATL1 | NM_080661 | 5.659145 |
| FLJ20054 | NM_019049 | 5.655552 |
| CCDC40 | AK000760 | 5.654316 |
| RASL12 | NM_016563 | 5.649811 |
| ADAMTS9 | NM_182920 | 5.620975 |
| KIAA1641 | NM_020970 | 5.617909 |
| KIF4A | NM_012310 | 5.609143 |
| ALMS1 | NM_015120 | 5.604125 |
| ZNF594 | AB058774 | 5.600441 |
| ZNF509 | NM_145291 | 5.596362 |
| E2F7 | NM_203394 | 5.583331 |
| COL15A1 | NM_001855 | 5.582902 |
| KCNMB1 | NM_004137 | 5.578849 |
| GUSBL2 | NM_206910 | 5.561197 |
| KIAA1333 | NM_017769 | 5.561125 |
| C21orf55 | NM_017833 | 5.557988 |
| RAB15 | NM_198686 | 5.557167 |
| RALA | NM_005402 | 5.556383 |
| FAM20A | NM_017565 | 5.556068 |
| IL6 | NM_000600 | 5.553272 |
| SYT15 | AK131036 | 5.542624 |
| LOXL4 | NM_032211 | 5.541165 |
| CAPS2 | AK091839 | 5.534377 |
| MRLC2 | NM_033546 | 5.520972 |
| NAV2 | AB063115 | 5.505185 |
| C20orf12 | NM_018152 | 5.503124 |
| TWSG1 | NM_020648 | 5.498013 |
| SORCS2 | NM_020777 | 5.493615 |
| PLCL1 | NM_006226 | 5.490436 |
| METTL7A | NM_014033 | 5.489893 |
| TMEM15 | NM_014908 | 5.487899 |
| EFNB2 | NM_004093 | 5.480056 |
| DST | NM_020388 | 5.478744 |
| UNQ338 | NM_001039792 | 5.475269 |
| RASSF2 | NM_014737 | 5.47242 |
| BCL2 | NM_000633 | 5.462879 |
| W95609 | W95609 | 5.455906 |
| N4BP2 | NM_018177 | 5.453676 |
| NEF3 | NM_005382 | 5.449026 |
| MYPN | NM_032578 | 5.434899 |
| PLEKHK1 | NM_145307 | 5.434366 |
| CARD9 | NM_052813 | 5.430511 |
| ANK3 | NM_020987 | 5.428845 |
| KRT77 | BC033366 | 5.423525 |
| RIN2 | NM_018993 | 5.417711 |
| ARL4A | NM_005738 | 5.40291 |
| FZD1 | NM_003505 | 5.399352 |
| EFCAB1 | NM_024593 | 5.387607 |
| FJX1 | NM_014344 | 5.381242 |
| SLFN5 | NM_144975 | 5.380752 |
| EFHB | NM_144715 | 5.377035 |
| MRPL43 | NM_176794 | 5.369209 |
| GRB14 | NM_004490 | 5.363715 |
| STX2 | NM_001980 | 5.363128 |
| FLJ39743 | NM_182562 | 5.343974 |
| PLXNA4A | AB046770 | 5.335409 |
| MYSM1 | AB067502 | 5.332049 |
| UACA | NM_001008224 | 5.331391 |
| NTNG1 | NM_014917 | 5.325998 |
| COL1A1 | Z74615 | 5.310894 |
| PITPNM3 | NM_031220 | 5.309837 |
| ANPEP | NM_001150 | 5.30472 |
| OGG1 | NM_016819 | 5.302408 |
| SLC26A3 | NM_000111 | 5.283227 |
| SPAG1 | NM_003114 | 5.270265 |
| CA2 | NM_000067 | 5.268714 |
| AGL | NM_000028 | 5.260731 |
| FZD7 | NM_003507 | 5.256572 |
| ST3GAL4 | AK021929 | 5.2553 |
| SLC8A3 | NM_183002 | 5.243694 |
| BAALC | NM_024812 | 5.24358 |
| NOV | NM_002514 | 5.241977 |
| C8orf4 | NM_020130 | 5.235127 |
| PXMP2 | NM_018663 | 5.230703 |
| ZNF75 | NM_007131 | 5.229447 |
| TRPA1 | NM_007332 | 5.225502 |
| GK | NM_203391 | 5.224342 |
| TMEM16A | NM_018043 | 5.216397 |
| ADRA2A | NM_000681 | 5.214985 |
| C10orf32 | NM_144591 | 5.207419 |
| DIAPH3 | NM_001042517 | 5.196032 |
| GAP43 | NM_002045 | 5.196011 |
| PRKD1 | NM_002742 | 5.191809 |
| DPT | NM_001937 | 5.189099 |
| GTF2I | NM_032999 | 5.186703 |
| FAM107B | NM_031453 | 5.18216 |
| MGC16121 | BC007360 | 5.178486 |
| RAB39B | NM_171998 | 5.166027 |
| ZNF658 | NM_033160 | 5.162715 |
| THSD4 | NM_024817 | 5.15613 |
| NTN4 | NM_021229 | 5.150871 |
| CTNND2 | NM_001332 | 5.144056 |
| ATP5E | NM_006886 | 5.141885 |
| MICALCL | NM_032867 | 5.141139 |
| IGFBP1 | NM_000596 | 5.141036 |
| UNQ9433 | NM_207413 | 5.141027 |
| UTY | NM_007125 | 5.138158 |
| FAM82A | AF435956 | 5.115836 |
| CTGF | NM_001901 | 5.106419 |
| TCF19 | BC033086 | 5.10156 |
| C9orf102 | NM_020207 | 5.093951 |
| DOPEY2 | NM_005128 | 5.086894 |
| TMEM26 | NM_178505 | 5.084661 |
| TPT1 | NM_003295 | 5.081889 |
| HS6ST3 | NM_153456 | 5.079982 |
| UCHL1 | NM_004181 | 5.0782 |
| APCDD1L | NM_153360 | 5.053529 |
| ROCK2 | NM_004850 | 5.052216 |
| ABHD11 | NM_031295 | 5.049108 |
| KBTBD3 | NM_198439 | 5.048657 |
| RAD9B | NM_152442 | 5.037381 |
| ZNF226 | NM_001032372 | 5.032882 |
| C4orf24 | NM_152618 | 5.032519 |
| FLJ39660 | AL834537 | 5.02967 |
| LYSMD3 | NM_198273 | 5.022911 |
| TRAF5 | NM_004619 | 5.021677 |
| SPINK1 | NM_003122 | 5.019425 |
| MCART2 | NM_001034172 | 5.010137 |
| NTRK2 | BX649001 | 5.009529 |
| FAM36A | NM_198076 | 5.007164 |
| STK39 | NM_013233 | 5.004107 |
| C10orf59 | NM_018363 | 4.98687 |
| ASNS | NM_001673 | 4.97433 |
| EDN1 | NM_001955 | 4.974048 |
| FLJ36748 | NM_152406 | 4.966995 |
| PANK3 | NM_024594 | 4.960677 |
| FLJ11151 | NM_018340 | 4.957288 |
| SLC38A2 | NM_018976 | 4.951681 |
| C6orf64 | NM_018322 | 4.949608 |
| HSPC157 | BC056896 | 4.948488 |
| CMKLR1 | NM_004072 | 4.946331 |
| CD48 | NM_001778 | 4.943369 |
| ITGBL1 | NM_004791 | 4.941274 |
| DISC1 | NM_018662 | 4.937805 |
| PAQR5 | NM_017705 | 4.933633 |
| MAP7 | NM_003980 | 4.920164 |
| EAF2 | NM_018456 | 4.918432 |
| BRCC3 | NM_001018055 | 4.913952 |
| SMG1 | NM_015092 | 4.913579 |
| C20orf42 | NM_017671 | 4.910607 |
| IGF2BP2 | NM_006548 | 4.901705 |
| SLC8A1 | BX648299 | 4.896049 |
| AP1S3 | NM_001039569 | 4.893179 |
| GOLPH4 | NM_014498 | 4.890756 |
| DLX1 | NM_178120 | 4.87879 |
| NRP1 | NM_003873 | 4.878458 |
| GPR161 | AK091271 | 4.877882 |
| CCNB2 | NM_004701 | 4.877874 |
| E2F1 | NM_005225 | 4.877254 |
| NETO2 | NM_018092 | 4.872388 |
| KIAA1529 | NM_020893 | 4.867044 |
| MSRB3 | NM_001031679 | 4.865029 |
| SURF5 | NM_133640 | 4.862387 |
| PFAS | NM_012393 | 4.859406 |
| CYP4X1 | NM_178033 | 4.849479 |
| LDHA | NM_005566 | 4.848881 |
| PHLDB1 | NM_015157 | 4.84653 |
| KPNA5 | NM_002269 | 4.83468 |
| NSBP1 | NM_030763 | 4.816972 |
| VNN3 | NM_018399 | 4.81319 |
| CLCN3 | NM_001829 | 4.80239 |
| ZNF596 | NM_173539 | 4.793527 |
| ASL | NM_000048 | 4.787983 |
| AGC1 | NM_013227 | 4.784642 |
| MS4A4A | NM_024021 | 4.784421 |
| KLHL5 | NM_015990 | 4.779948 |
| TMEM49 | BC024020 | 4.779747 |
| RAB3D | BC007960 | 4.765474 |
| FLJ14167 | AK024229 | 4.759268 |
| EXOC7 | NM_001013839 | 4.755394 |
| C14orf62 | NR_001459 | 4.753665 |
| MFI2 | NM_033316 | 4.753403 |
| PTPN11 | NM_002834 | 4.752155 |
| FLJ39370 | NM_152400 | 4.749259 |
| C10orf110 | NM_018470 | 4.749129 |
| ACOT4 | NM_152331 | 4.748909 |
| CBS | NM_000071 | 4.742471 |
| SELENBP1 | NM_003944 | 4.739523 |
| EIF2C1 | NM_012199 | 4.737277 |
| FGL2 | NM_006682 | 4.732436 |
| PTPRO | NM_030667 | 4.731195 |
| HMGA2 | NM_003483 | 4.730671 |
| SP140 | NM_007237 | 4.725354 |
| KCNK17 | NM_031460 | 4.711209 |
| LMLN | AL832783 | 4.708558 |
| CCDC81 | NM_021827 | 4.700601 |
| GALNT1 | NM_020474 | 4.697416 |
| PHTF1 | NM_006608 | 4.697408 |
| EBF3 | NM_001005463 | 4.693155 |
| PHCA | NM_018367 | 4.684621 |
| C7orf25 | NM_024054 | 4.68441 |
| SMCY | NM_004653 | 4.683913 |
| NLGN1 | NM_014932 | 4.675627 |
| ZNF439 | NM_152262 | 4.675614 |
| MGC23985 | NM_206966 | 4.660983 |
| FZD3 | NM_017412 | 4.658562 |
| FLJ35024 | AF424541 | 4.653758 |
| PLAC8 | NM_016619 | 4.651148 |
| MND1 | NM_032117 | 4.645475 |
| TMLHE | NM_018196 | 4.643776 |
| NARG2 | NM_024611 | 4.642993 |
| EFCAB2 | NM_032328 | 4.638702 |
| ITCH | NM_031483 | 4.638342 |
| KIAA1199 | NM_018689 | 4.634357 |
| GAS1 | NM_002048 | 4.634081 |
| CKAP2L | NM_152515 | 4.633691 |
| DEPDC1 | NM_017779 | 4.628241 |
| C10orf13 | NM_152429 | 4.627272 |
| C9orf19 | NM_022343 | 4.623474 |
| MARVELD2 | NM_144724 | 4.618427 |
| C18orf1 | NM_181482 | 4.618023 |
| DRD1 | NM_000794 | 4.616151 |
| THBS2 | L12350 | 4.61373 |
| FN1 | NM_212482 | 4.612983 |
| OLFML2A | NM_182487 | 4.608737 |
| PTPN13 | NM_080685 | 4.603941 |
| FNDC1 | NM_032532 | 4.593122 |
| PAIP1 | NM_006451 | 4.592368 |
| CIT | NM_007174 | 4.58639 |
| CTAGE1 | NM_172241 | 4.582617 |
| MCAM | NM_006500 | 4.581832 |
| SNTB1 | AF028828 | 4.57999 |
| ITIH5 | NM_030569 | 4.576947 |
| DEADC1 | NM_182503 | 4.564462 |
| FOS | NM_005252 | 4.562067 |
| TTC3 | NM_003316 | 4.560661 |
| DSCR1L2 | NM_013441 | 4.54979 |
| WWC2 | NM_024949 | 4.53542 |
| FGF7 | NM_002009 | 4.532794 |
| AGTR1 | NM_031850 | 4.530303 |
| CCNK | NM_003858 | 4.526387 |
| XLKD1 | NM_006691 | 4.525084 |
| TTK | NM_003318 | 4.524613 |
| KIAA0802 | BC040542 | 4.524549 |
| TRIM34 | NM_130390 | 4.52165 |
| HCG18 | CR606587 | 4.520114 |
| NIPSNAP3B | NM_018376 | 4.517275 |
| MANSC1 | NM_018050 | 4.516392 |
| DDAH1 | NM_012137 | 4.515926 |
| STK17A | NM_004760 | 4.514475 |
| DKFZp667E0512 | AL713660 | 4.509527 |
| TMSL3 | NM_183049 | 4.508775 |
| NEFH | NM_021076 | 4.506025 |
| PPP1R14A | NM_033256 | 4.50262 |
| SNCAIP | NM_005460 | 4.501479 |
| F3 | NM_001993 | 4.501058 |
| IFT81 | NM_031473 | 4.494739 |
| SMCHD1 | AB014550 | 4.494724 |
| ZNF287 | NM_020653 | 4.492475 |
| LUZP5 | AK000318 | 4.482536 |
| PBK | NM_018492 | 4.481316 |
| CTPS | NM_001905 | 4.479688 |
| FAM82B | NM_016033 | 4.479656 |
| PVRL3 | NM_015480 | 4.475293 |
| ERCC8 | NM_001007233 | 4.474993 |
| C6orf168 | NM_032511 | 4.47272 |
| B4GALT6 | NM_004775 | 4.468172 |
| CXorf43 | NM_144657 | 4.467496 |
| PAX9 | U59628 | 4.46473 |
| FADD | NM_003824 | 4.461388 |
| ABRA | NM_139166 | 4.459478 |
| TMTC1 | NM_175861 | 4.45497 |
| ELAC1 | NM_018696 | 4.453977 |
| SLC16A14 | NM_152527 | 4.450604 |
| SSBP1 | NM_003143 | 4.448487 |
| TBC1D1 | NM_015173 | 4.433903 |
| CMAH | NR_002174 | 4.429072 |
| C20orf103 | NM_012261 | 4.426973 |
| ASCIZ | NM_015251 | 4.423204 |
| TIAM2 | NM_012454 | 4.419012 |
| MEG3 | AK098378 | 4.417435 |
| CCDC112 | NM_152549 | 4.414966 |
| EXTL2 | NM_001439 | 4.406581 |
| ZFP90 | NM_133458 | 4.399193 |
| RABIF | NM_002871 | 4.398035 |
| ARSJ | NM_024590 | 4.396151 |
| FNTA | AB209689 | 4.393021 |
| NANOS1 | NM_199461 | 4.392713 |
| TBC1D19 | NM_018317 | 4.385716 |
| DMD | NM_004010 | 4.385493 |
| C9orf18 | NM_198469 | 4.382205 |
| SLC4A7 | NM_003615 | 4.381077 |
| CD59 | NM_203330 | 4.375617 |
| ZNF435 | NM_025231 | 4.372831 |
| C1QTNF3 | NM_181435 | 4.361701 |
| KIAA1908 | AB067495 | 4.360794 |
| FRMPD4 | NM_014728 | 4.355893 |
| XRCC2 | CR749256 | 4.355287 |
| P2RY6 | NM_176798 | 4.347882 |
| EDNRA | NM_001957 | 4.345083 |
| PGPEP1 | NM_017712 | 4.340968 |
| AHRR | NM_020731 | 4.330821 |
| PLOD2 | NM_182943 | 4.325977 |
| RPS26 | NM_001029 | 4.322187 |
| IQCG | NM_032263 | 4.320739 |
| MGC70863 | AL365511 | 4.318049 |
| KIAA1644 | AB051431 | 4.312984 |
| C6orf32 | AB002384 | 4.312185 |
| TSPAN9 | NM_006675 | 4.30982 |
| ZNF568 | BC016334 | 4.309553 |
| NOSTRIN | NM_052946 | 4.30905 |
| LDB2 | NM_001290 | 4.305818 |
| RUVBL1 | NM_003707 | 4.30152 |
| TMEM19 | NM_018279 | 4.299981 |
| RPL26 | NM_000987 | 4.298381 |
| TRIM15 | NM_033229 | 4.296207 |
| FLJ42709 | BC044619 | 4.295794 |
| PCK2 | NM_004563 | 4.295212 |
| C20orf133 | NM_001033086 | 4.292597 |
| MAMDC2 | NM_153267 | 4.290952 |
| ZNF462 | NM_021224 | 4.288918 |
| FLJ36492 | NM_182568 | 4.288423 |
| NT5E | NM_002526 | 4.285704 |
| FGFR3 | NM_000142 | 4.284268 |
| ZNF514 | NM_032788 | 4.283931 |
| TGFBI | NM_000358 | 4.277698 |
| FLJ10292 | NM_018048 | 4.27153 |
| PXDN | AF200348 | 4.269365 |
| LOH3CR2A | NM_013343 | 4.264992 |
| FGF14 | NM_175929 | 4.264971 |
| EME2 | AK074080 | 4.263179 |
| KIAA1212 | NM_018084 | 4.260431 |
| VIM | NM_003380 | 4.260181 |
| PAFAH1B1 | NM_000430 | 4.259975 |
| PCNT | NM_006031 | 4.255266 |
| UBE2C | NM_181803 | 4.253785 |
| ADARB1 | NM_015833 | 4.251859 |
| NANOG | NM_024865 | 4.24699 |
| DGKG | NM_001346 | 4.246268 |
| SAMD13 | NM_001010971 | 4.246151 |
| ICK | NM_016513 | 4.245048 |
| ZNF577 | NM_032679 | 4.245011 |
| SLC28A2 | NM_004212 | 4.244808 |
| TCHP | NM_032300 | 4.241922 |
| ZNF682 | AK074843 | 4.241711 |
| ERRFI1 | NM_018948 | 4.239087 |
| KIAA0408 | NM_014702 | 4.238307 |
| RPL31 | NM_000993 | 4.237952 |
| SUSD2 | NM_019601 | 4.237816 |
| EPHA5 | BX537946 | 4.232818 |
| LCORL | AL133031 | 4.232118 |
| DKFZp761I2123 | NM_031449 | 4.231996 |
| OLFML3 | NM_020190 | 4.224225 |
| KIAA1524 | AB040957 | 4.224204 |
| GSG2 | AK056691 | 4.22304 |
| FLJ20323 | AL136892 | 4.217904 |
| PAQR8 | NM_133367 | 4.214567 |
| SEMA3A | NM_006080 | 4.213504 |
| PPIG | NM_004792 | 4.20561 |
| SLC26A1 | NM_134425 | 4.205542 |
| C1orf112 | NM_018186 | 4.202449 |
| SNAP29 | NM_004782 | 4.198838 |
| FABP5 | NM_001444 | 4.196094 |
| MARCKS | NM_002356 | 4.194092 |
| RPLP1 | NM_001003 | 4.192904 |
| TRIM23 | NM_001656 | 4.190715 |
| RIMS3 | NM_014747 | 4.189499 |
| FLJ12688 | AB051533 | 4.18796 |
| IL11 | NM_000641 | 4.182327 |
| FAM64A | NM_019013 | 4.181824 |
| PCSK1 | NM_000439 | 4.177218 |
| BTG3 | BC028229 | 4.175212 |
| CDKL5 | NM_003159 | 4.173752 |
| C6orf162 | NM_020425 | 4.167981 |
| TSPAN15 | NM_012339 | 4.167124 |
| C8orf38 | NM_152416 | 4.164877 |
| SLC16A12 | NM_213606 | 4.164321 |
| U66046 | U66046 | 4.162901 |
| HOXC8 | NM_022658 | 4.160615 |
| DGKI | NM_004717 | 4.160438 |
| FGD4 | NM_139241 | 4.159399 |
| ZNF187 | NM_152736 | 4.158839 |
| UROS | NM_000375 | 4.158492 |
| FAM41C | BC047940 | 4.156431 |
| DKFZP564O0823 | NM_015393 | 4.155141 |
| SLIT3 | NM_003062 | 4.155082 |
| FLJ21075 | NM_025031 | 4.151608 |
| ICMT | NM_012405 | 4.151542 |
| AHNAK | NM_001620 | 4.15077 |
| ZRANB3 | BC064616 | 4.149039 |
| IFT74 | NM_025103 | 4.147959 |
| MYL6 | NM_079423 | 4.136277 |
| FAM86A | NM_201400 | 4.135075 |
| ACAD9 | NM_014049 | 4.125443 |
| MAP2 | NM_002374 | 4.119768 |
| CAV1 | NM_001753 | 4.115549 |
| PTPLA | NM_014241 | 4.110801 |
| C4orf25 | NM_173660 | 4.107428 |
| PLA2G4C | NM_003706 | 4.101471 |
| LHX2 | NM_004789 | 4.100981 |
| TARBP1 | NM_005646 | 4.096912 |
| AFF2 | NM_002025 | 4.09632 |
| C1QTNF7 | NM_031911 | 4.092788 |
| MPDZ | AB210041 | 4.090877 |
| CRSP9 | NM_004270 | 4.086017 |
| C9orf103 | NM_001001551 | 4.084883 |
| KATNAL2 | AL512748 | 4.081308 |
| FLJ30294 | NM_144632 | 4.08106 |
| C20orf166 | NM_178463 | 4.078678 |
| ECM2 | BC036806 | 4.076184 |
| ALDH1A1 | NM_000689 | 4.075551 |
| KIAA1586 | NM_020931 | 4.070768 |
| EPB41L3 | NM_012307 | 4.069261 |
| CDKN2A | NM_058197 | 4.064633 |
| GPD1L | NM_015141 | 4.064579 |
| CSS3 | NM_175856 | 4.063931 |
| RGN | NM_004683 | 4.059196 |
| PODXL | NM_005397 | 4.056117 |
| DKFZP586H2123 | NM_015430 | 4.053213 |
| FAM83D | NM_030919 | 4.052448 |
| LRRC17 | NM_005824 | 4.048272 |
| HIG2 | NM_013332 | 4.043087 |
| CCDC46 | NM_145036 | 4.032598 |
| CATSPER1 | NM_053054 | 4.032572 |
| APC | NM_000038 | 4.031902 |
| LRRC34 | NM_153353 | 4.024916 |
| ANKRD12 | NM_015208 | 4.023018 |
| C6orf199 | NM_145025 | 4.018782 |
| FKSG2 | NM_021631 | 4.018427 |
| TANC2 | AK021886 | 4.012866 |
| XK | NM_021083 | 4.008727 |
| ADCY4 | NM_139247 | 4.005713 |
| CDH8 | NM_001796 | 4.00476 |
| LIMS3 | BC094740 | 4.000812 |
| CENPM | NM_001002876 | 3.999781 |
| ARHGDIB | NM_001175 | 3.994919 |
| FBXO36 | BC022538 | 3.986835 |
| BCR | NM_021574 | 3.985809 |
| TMEM14A | NM_014051 | 3.985198 |
| GPNMB | NM_001005340 | 3.983044 |
| CPSF3 | NM_016207 | 3.979693 |
| MIER1 | NM_020948 | 3.979483 |
| MNS1 | NM_018365 | 3.971528 |
| PDE5A | NM_001083 | 3.969691 |
| TMTC4 | NM_032813 | 3.96882 |
| CUL5 | BG031574 | 3.967347 |
| HBLD2 | NM_030940 | 3.967066 |
| SECTM1 | NM_003004 | 3.966954 |
| HSD17B12 | BC012536 | 3.962737 |
| ACTG1 | NM_001614 | 3.960907 |
| RTN4IP1 | NM_032730 | 3.955099 |
| AGPAT4 | NM_001012733 | 3.945643 |
| KIAA1704 | NM_018559 | 3.944416 |
| HLA-DPA1 | NM_033554 | 3.94314 |
| KCNAB2 | NM_003636 | 3.940687 |
| LRBA | NM_006726 | 3.940056 |
| DPF3 | AK124946 | 3.937857 |
| SLC43A2 | NM_152346 | 3.930112 |
| EML4 | NM_019063 | 3.9268 |
| DYNLL1 | NM_003746 | 3.922539 |
| CENTA2 | NM_018404 | 3.922141 |
| RGS7 | NM_002924 | 3.917754 |
| RAD54L | NM_003579 | 3.916002 |
| FEZ2 | NM_005102 | 3.9105 |
| FLJ32745 | NM_144978 | 3.909514 |
| HHIP | NM_022475 | 3.908211 |
| UTP15 | NM_032175 | 3.905166 |
| PTHB1 | NM_198428 | 3.904458 |
| TMEM117 | NM_032256 | 3.903999 |
| ZNF613 | AK023652 | 3.90316 |
| FLJ12993 | XM_496707 | 3.900689 |
| GTF2H5 | NM_207118 | 3.894567 |
| DUSP18 | NM_152511 | 3.89338 |
| CD46 | NM_002389 | 3.893114 |
| KGFLP1 | NM_174950 | 3.892643 |
| MEGF6 | NM_001409 | 3.892088 |
| FOXL2 | NM_023067 | 3.89126 |
| C8orf58 | NM_001013842 | 3.888955 |
| RASSF8 | NM_007211 | 3.888161 |
| CDC2 | NM_001786 | 3.886918 |
| U52054 | U52054 | 3.881799 |
| PLCL2 | NM_015184 | 3.874457 |
| PLEKHG1 | NM_001029884 | 3.871478 |
| FLJ20097 | NM_017667 | 3.869816 |
| PGBD1 | NM_032507 | 3.869308 |
| SCRG1 | NM_007281 | 3.869278 |
| C1orf53 | NM_001024594 | 3.868904 |
| LGI2 | NM_018176 | 3.868304 |
| AMIGO2 | NM_181847 | 3.867138 |
| CAPZA2 | NM_006136 | 3.866747 |
| DPYD | NM_000110 | 3.863557 |
| PGK1 | NM_000291 | 3.861354 |
| FAM121A | NM_198450 | 3.85988 |
| AVIL | NM_006576 | 3.849125 |
| C14orf143 | NM_145231 | 3.848687 |
| C5orf5 | NM_016603 | 3.84698 |
| PLA2G2E | NM_014589 | 3.845215 |
| SCD5 | NM_001037582 | 3.841754 |
| WDR69 | NM_178821 | 3.840905 |
| FOXM1 | NM_202002 | 3.840392 |
| PRRX2 | NM_016307 | 3.833339 |
| SERPINI1 | NM_005025 | 3.832447 |
| COL1A2 | NM_000089 | 3.832116 |
| CCDC91 | NM_018318 | 3.829099 |
| BAG2 | NM_004282 | 3.828274 |
| ARNTL2 | AF256215 | 3.82766 |
| MUC20 | NM_152673 | 3.827586 |
| PDCD5 | NM_004708 | 3.826988 |
| AGMAT | NM_024758 | 3.824624 |
| FAM7A3 | XM_930891 | 3.823992 |
| EPS8L1 | NM_133180 | 3.822434 |
| ROPN1B | NM_001012337 | 3.819666 |
| SLC44A3 | NM_152369 | 3.819344 |
| FLJ39653 | NM_152684 | 3.81872 |
| RP11-151A6.2 | BC001077 | 3.817051 |
| WDR19 | NM_025132 | 3.814401 |
| GZMK | NM_002104 | 3.810696 |
| FAIM | NM_018147 | 3.810195 |
| MELK | NM_014791 | 3.805614 |
| TRIM36 | NM_018700 | 3.80501 |
| FLJ40432 | NM_152523 | 3.803765 |
| CETN3 | NM_004365 | 3.802903 |
| FBXO4 | NM_012176 | 3.798419 |
| C10orf63 | NM_145010 | 3.784979 |
| LMOD1 | NM_012134 | 3.784979 |
| C3orf34 | NM_032898 | 3.783963 |
| SGCD | NM_000337 | 3.783068 |
| GRIA3 | NM_000828 | 3.782609 |
| PHF6 | NM_032335 | 3.781703 |
| FLJ31033 | AK023743 | 3.780909 |
| MYO5A | NM_000259 | 3.780708 |
| MYH2 | NM_017534 | 3.776833 |
| NEK2 | NM_002497 | 3.775833 |
| PARP4 | NM_006437 | 3.775832 |
| FAM38B | NM_022068 | 3.775298 |
| MBNL3 | AK095439 | 3.773914 |
| TRAF3IP3 | AK022798 | 3.773683 |
| MAP1D | NM_199227 | 3.771821 |
| KIAA2002 | AB082533 | 3.771673 |
| ACTG2 | NM_001615 | 3.77007 |
| EPPB9 | NM_015681 | 3.765234 |
| MAP1A | NM_002373 | 3.763855 |
| HRBL | NM_006076 | 3.761812 |
| ZNF17 | AB075827 | 3.759827 |
| BST1 | NM_004334 | 3.759694 |
| ARHGAP23 | XM_930977 | 3.75575 |
| KIAA0831 | NM_014924 | 3.755445 |
| TMEM67 | NM_153704 | 3.753679 |
| GPR112 | NM_153834 | 3.753117 |
| SIRT5 | NM_031244 | 3.75216 |
| C11orf65 | NM_152587 | 3.74901 |
| TIMP3 | AB051444 | 3.747461 |
| SPON1 | NM_006108 | 3.747018 |
| C11orf63 | NM_024806 | 3.746631 |
| CRIP1 | NM_001311 | 3.746011 |
| FGD5 | NM_152536 | 3.743631 |
| DCBLD2 | NM_080927 | 3.742934 |
| ZFYVE9 | NM_004799 | 3.742793 |
| CASD1 | NM_022900 | 3.738689 |
| CENPN | AK023669 | 3.735758 |
| TTR | NM_000371 | 3.734189 |
| ENC1 | NM_003633 | 3.734126 |
| DNMT2 | NM_004412 | 3.732934 |
| EDN2 | NM_001956 | 3.725964 |
| RP11-301I17.1 | NM_017993 | 3.722942 |
| TAS2R50 | NM_176890 | 3.721294 |
| SLC16A3 | NM_004207 | 3.720613 |
| OLFM1 | NM_006334 | 3.720338 |
| KCNJ6 | NM_002240 | 3.719168 |
| PSCD4 | NM_013385 | 3.715737 |
| SYNE1 | NM_182961 | 3.707023 |
| LYZ | NM_000239 | 3.705463 |
| ALDH1L2 | CR749561 | 3.704871 |
| TSLP | NM_033035 | 3.703477 |
| MSL3L1 | NM_078628 | 3.702135 |
| ADIPOQ | NM_004797 | 3.701228 |
| CENPI | NM_006733 | 3.701074 |
| ADFP | NM_001122 | 3.693975 |
| VIT | NM_053276 | 3.692739 |
| IGF2BP1 | NM_006546 | 3.68758 |
| FLJ23577 | NM_024867 | 3.684515 |
| MICAL-L2 | NM_182924 | 3.678018 |
| MGC33212 | NM_152773 | 3.677057 |
| ZNF528 | NM_032423 | 3.672964 |
| DYRK2 | NM_006482 | 3.671912 |
| FLJ11171 | NM_018348 | 3.671017 |
| MDGA1 | NM_153487 | 3.668883 |
| NAT12 | NM_001011713 | 3.667654 |
| MTHFD2L | NM_001004346 | 3.667321 |
| ENDOGL1 | NM_005107 | 3.665799 |
| CYP4F12 | NM_023944 | 3.661611 |
| CALM2 | NM_001743 | 3.661342 |
| C8orf70 | NM_016010 | 3.659986 |
| DUSP5P | NR_002834 | 3.65996 |
| SRP9 | NM_003133 | 3.658951 |
| TTC12 | NM_017868 | 3.657528 |
| KIAA1443 | NM_020834 | 3.655518 |
| MARS | NM_004990 | 3.653133 |
| HSPA8 | NM_006597 | 3.653088 |
| COMMD6 | NM_203497 | 3.650555 |
| DHX34 | D50924 | 3.650218 |
| ZMYM4 | NM_005095 | 3.64868 |
| HOXD10 | NM_002148 | 3.648233 |
| VCX | NM_013452 | 3.647613 |
| STS-1 | NM_032873 | 3.647282 |
| KLF12 | NM_016285 | 3.645892 |
| SYCP2 | NM_014258 | 3.644241 |
| DIP13B | NM_018171 | 3.642517 |
| NDN | NM_002487 | 3.641317 |
| HEMK1 | NM_016173 | 3.634775 |
| LGICZ1 | NM_180990 | 3.633508 |
| ACTA1 | NM_001100 | 3.633461 |
| ZC3H7B | NM_017590 | 3.632448 |
| CDC23 | BC010944 | 3.629492 |
| C6orf51 | NM_138408 | 3.62903 |
| THEM2 | NM_018473 | 3.627577 |
| C9orf58 | NM_001002260 | 3.626383 |
| ELTD1 | BC025721 | 3.626366 |
| DJ122O8.2 | NM_020466 | 3.623741 |
| ANKRD42 | NM_182603 | 3.615668 |
| FKBP3 | NM_002013 | 3.613517 |
| ELA3B | NM_007352 | 3.61225 |
| FLJ23191 | NM_024574 | 3.611656 |
| ZNF624 | NM_020787 | 3.610551 |
| OMD | NM_005014 | 3.605391 |
| CKLF | NM_016951 | 3.603489 |
| CHST2 | NM_004267 | 3.603234 |
| HLCS | NM_000411 | 3.594533 |
| OAZ3 | NM_016178 | 3.594495 |
| KIAA1456 | NM_020844 | 3.590132 |
| C14orf168 | NM_031427 | 3.58731 |
| SMUG1 | AK091468 | 3.582506 |
| ARHGEF6 | NM_004840 | 3.581949 |
| PDE4D | BC008390 | 3.581046 |
| AP2B1 | NM_001282 | 3.579036 |
| CCDC99 | NM_017785 | 3.57118 |
| LZTS1 | NM_021020 | 3.570508 |
| CDH11 | NM_001797 | 3.569365 |
| RAB42 | NM_152304 | 3.565466 |
| ADRA2C | NM_000683 | 3.565433 |
| SGOL1 | NM_001012409 | 3.564109 |
| RBP4 | NM_006744 | 3.562632 |
| ZCRB1 | NM_033114 | 3.561388 |
| STK32B | NM_018401 | 3.559193 |
| C3orf58 | NM_173552 | 3.559124 |
| CTAGE5 | AF338234 | 3.556159 |
| ZNF33B | NM_006955 | 3.553918 |
| MKRN3 | NM_005664 | 3.547574 |
| C1GALT1 | NM_020156 | 3.547508 |
| ENY2 | AK095651 | 3.544771 |
| EHD2 | NM_014601 | 3.541447 |
| GFRA1 | NM_005264 | 3.541161 |
| PRRT3 | NM_207351 | 3.540154 |
| SHRM | NM_020859 | 3.5395 |
| SLC26A4 | NM_000441 | 3.536904 |
| TXNL5 | NM_032731 | 3.535052 |
| PHTF2 | NM_020432 | 3.533922 |
| SUSD1 | NM_022486 | 3.531014 |
| MLH3 | NM_014381 | 3.530537 |
| ANGPTL1 | NM_004673 | 3.52888 |
| C1orf110 | BC040018 | 3.524432 |
| DOCK7 | NM_033407 | 3.522873 |
| DMRTC1 | AJ291670 | 3.522762 |
| LAMA2 | NM_000426 | 3.521129 |
| LRRC2 | NM_024512 | 3.517608 |
| RGMB | NM_001012761 | 3.517338 |
| CCPG1 | NM_020739 | 3.517163 |
| TP73L | NM_003722 | 3.51546 |
| MAPK10 | NM_138980 | 3.515205 |
| SEC63D1 | NM_198550 | 3.514602 |
| PPIL6 | NM_173672 | 3.511356 |
| ATP10D | NM_020453 | 3.511162 |
| ARHGEF9 | NM_015185 | 3.510025 |
| RABL3 | NM_173825 | 3.509629 |
| DLX4 | NM_138281 | 3.509031 |
| C20orf29 | NM_018347 | 3.506178 |
| HORMAD2 | NM_152510 | 3.506177 |
| FGF9 | NM_002010 | 3.50607 |
| TTYH2 | BC021035 | 3.50506 |
| FANCD2 | NM_001018115 | 3.502029 |
| C5orf21 | NM_032042 | 3.501512 |
| C6orf65 | NM_152731 | 3.50112 |
| ZBED3 | NM_032367 | 3.500762 |
| PPP3CA | NM_000944 | 3.500517 |
| WHSC1 | NM_007331 | 3.500442 |
| FHAD1 | AK093883 | 3.500316 |
| ERICH1 | NM_207332 | 3.500117 |
| C6orf173 | NM_001012507 | 3.499149 |
| RUNDC1 | NM_173079 | 3.498758 |
| FAM72A | NM_207418 | 3.497611 |
| PON2 | NM_000305 | 3.497332 |
| RGC32 | NM_014059 | 3.49521 |
| CASC1 | NM_018272 | 3.494772 |
| TSNAXIP1 | NM_018430 | 3.493664 |
| PCSK7 | NM_004716 | 3.492741 |
| ACADL | NM_001608 | 3.490956 |
| TNFSF4 | NM_003326 | 3.490403 |
| PFTK1 | NM_012395 | 3.489601 |
| JDP2 | NM_130469 | 3.486749 |
| MT1M | NM_176870 | 3.485478 |
| ADAM28 | NM_021777 | 3.485351 |
| UNQ6490 | AY358209 | 3.48468 |
| FMNL3 | NM_175736 | 3.482608 |
| RP11-298P3.3 | NM_033111 | 3.480467 |
| PITPNC1 | AK094724 | 3.479518 |
| PLEKHA8 | NM_032639 | 3.479488 |
| OBFC2A | NM_001031716 | 3.478047 |
| SPAG16 | NM_024532 | 3.477077 |
| NRBP2 | NM_178564 | 3.474104 |
| DNAJC10 | NM_018981 | 3.472864 |
| FLJ31438 | NM_152385 | 3.471535 |
| DEPDC4 | NM_152317 | 3.470103 |
| C1orf19 | NM_052965 | 3.469482 |
| FAM11B | NR_000034 | 3.469441 |
| DUSP22 | NM_020185 | 3.469429 |
| STAC | NM_003149 | 3.463706 |
| CHMP5 | NM_016410 | 3.463515 |
| TRPV2 | NM_016113 | 3.462711 |
| ZNF33A | NM_006974 | 3.462706 |
| RAP1GDS1 | NM_021159 | 3.458857 |
| MTERFD3 | NM_025198 | 3.458649 |
| BCL2L11 | NM_138622 | 3.45625 |
| KIAA0467 | NM_015284 | 3.455373 |
| CELSR1 | NM_014246 | 3.454571 |
| CXCL14 | NM_004887 | 3.45343 |
| PSEN2 | NM_000447 | 3.452735 |
| DEPDC6 | NM_022783 | 3.452231 |
| C6orf194 | NM_001007531 | 3.451906 |
| CAP2 | NM_006366 | 3.449785 |
| C18orf18 | BC010538 | 3.449021 |
| DRB1 | NM_152945 | 3.448146 |
| CBWD3 | AF293368 | 3.447603 |
| VIL2 | NM_003379 | 3.445108 |
| HSPA12A | AB007877 | 3.441924 |
| KCTD8 | NM_198353 | 3.441649 |
| NAP5 | AK124659 | 3.439508 |
| DYNLT1 | NM_006519 | 3.438868 |
| ZNF585A | AK123296 | 3.43748 |
| EDNRB | NM_003991 | 3.434863 |
| C8orf59 | BC032347 | 3.427673 |
| ARHGAP28 | NM_001010000 | 3.424526 |
| OGT | NM_181672 | 3.423253 |
| UBE2D4 | NM_015983 | 3.420538 |
| FER | NM_005246 | 3.419544 |
| CRIM1 | NM_016441 | 3.418339 |
| CLUAP1 | NM_024793 | 3.417924 |
| C21orf81 | NM_153750 | 3.417318 |
| ACRC | NM_052957 | 3.41703 |
| SGCA | NM_000023 | 3.415246 |
| SC4MOL | AV701505 | 3.414701 |
| PARP11 | NM_020367 | 3.413054 |
| PLXNA2 | AK055196 | 3.411461 |
| MARK1 | NM_018650 | 3.409857 |
| PSKH1 | NM_006742 | 3.408237 |
| HSF2BP | NM_007031 | 3.406672 |
| SEC22C | NM_032970 | 3.406561 |
| TSPAN12 | NM_012338 | 3.406362 |
| DISP1 | NM_032890 | 3.406105 |
| SCG5 | NM_003020 | 3.405853 |
| ANKRD45 | NM_198493 | 3.404203 |
| GSTT2 | NM_000854 | 3.402444 |
| EPPK1 | NM_031308 | 3.40175 |
| CPE | NM_001873 | 3.399921 |
| DOC1 | NM_182909 | 3.399856 |
| CTDSP1 | NM_021198 | 3.397499 |
| FBXL16 | NM_153350 | 3.396575 |
| MYADM | NM_001020818 | 3.395625 |
| SOST | NM_025237 | 3.394117 |
| UCHL3 | NM_006002 | 3.3932 |
| RUFY3 | NM_001037442 | 3.392393 |
| AGBL2 | NM_024783 | 3.390441 |
| HIST1H4C | NM_003542 | 3.389699 |
| DYNC2LI1 | NM_015522 | 3.389576 |
| ASRGL1 | BC006267 | 3.388917 |
| C5 | NM_001735 | 3.387704 |
| BACH2 | NM_021813 | 3.386437 |
| WNT5A | NM_003392 | 3.386113 |
| KRT25 | NM_181534 | 3.385832 |
| ESM1 | NM_007036 | 3.384118 |
| CAV3 | NM_001234 | 3.381959 |
| DDEF1IT1 | NR_002765 | 3.381689 |
| COVA1 | NM_182314 | 3.380718 |
| ACADM | NM_000016 | 3.380135 |
| KIFC1 | NM_002263 | 3.376431 |
| CXorf23 | NM_198279 | 3.375038 |
| FRZB | NM_001463 | 3.374387 |
| DKFZP564C152 | AL049980 | 3.373105 |
| GPM6B | NM_001001996 | 3.37289 |
| SPATA22 | NM_032598 | 3.370869 |
| SFRS12 | AB209694 | 3.369079 |
| CCL13 | NM_005408 | 3.367879 |
| HHAT | NM_018194 | 3.366053 |
| BAAT | NM_001701 | 3.361106 |
| TRAM1L1 | NM_152402 | 3.360997 |
| COP1 | NM_001017534 | 3.360778 |
| GMPPB | NM_021971 | 3.360697 |
| IL17B | NM_014443 | 3.36032 |
| PB1 | NM_181042 | 3.357653 |
| WFDC8 | NM_130896 | 3.35576 |
| ID2 | NM_002166 | 3.355468 |
| FLG | NM_002016 | 3.354304 |
| PRDM11 | AK097878 | 3.351902 |
| PDE1C | NM_005020 | 3.346237 |
| EPDR1 | NM_017549 | 3.344258 |
| POLB | NM_002690 | 3.343272 |
| NPAL2 | AK025015 | 3.341178 |
| SULT1A1 | NM_177529 | 3.34056 |
| SMYD3 | NM_022743 | 3.337546 |
| CDC42BPA | NM_014826 | 3.332245 |
| MGC50559 | NM_173802 | 3.328483 |
| LYSMD4 | NM_152449 | 3.328335 |
| SF3A1 | NM_005877 | 3.324518 |
| FLJ30655 | NM_144643 | 3.32341 |
| ABCB1 | NM_000927 | 3.322249 |
| VCAM1 | NM_001078 | 3.321648 |
| CCT6B | NM_006584 | 3.321166 |
| RCBTB2 | NM_001268 | 3.320937 |
| WDR51A | NM_015426 | 3.319832 |
| FMN1 | BC029107 | 3.319104 |
| RABGAP1L | NM_014857 | 3.319008 |
| C13orf3 | BC013418 | 3.318802 |
| ALF | NM_172196 | 3.31773 |
| RAB33A | NM_004794 | 3.317261 |
| GNG11 | NM_004126 | 3.314597 |
| C1orf135 | NM_024037 | 3.311061 |
| ZNF655 | NM_001009956 | 3.310935 |
| C11orf71 | NM_019021 | 3.309199 |
| LGALS2 | NM_006498 | 3.30763 |
| NPHP1 | NM_000272 | 3.307606 |
| DKFZp686I1569 | NM_001024596 | 3.302004 |
| CSPG4 | NM_001897 | 3.301269 |
| MYO1B | NM_012223 | 3.300697 |
| FAM121B | NM_024122 | 3.299372 |
| ROPN1 | NM_017578 | 3.298235 |
| GNG2 | NM_053064 | 3.296823 |
| SEC11L3 | NM_033280 | 3.296263 |
| ALG1 | NM_019109 | 3.295271 |
| ZCCHC5 | NM_152694 | 3.294173 |
| WDR31 | NM_001012361 | 3.292191 |
| C4orf22 | NM_152770 | 3.291726 |
| POLI | NM_007195 | 3.288191 |
| ORC6L | NM_014321 | 3.286742 |
| MCCC1 | NM_020166 | 3.284635 |
| ZBTB38 | AK094201 | 3.282428 |
| ANKRD50 | NM_020337 | 3.280568 |
| TAF13 | NM_005645 | 3.277291 |
| C5orf24 | NM_152409 | 3.277018 |
| CD163L1 | NM_174941 | 3.276005 |
| ZBED5 | NM_021211 | 3.274972 |
| KIAA0773 | NM_014690 | 3.274056 |
| FAM33A | NM_182620 | 3.273918 |
| CDH13 | NM_001257 | 3.268827 |
| KIAA1549 | AL136736 | 3.266609 |
| FANCM | NM_020937 | 3.264965 |
| C14orf126 | NM_080664 | 3.263589 |
| ARPM1 | NM_032487 | 3.261978 |
| RAFTLIN | NM_015150 | 3.2603 |
| RHOJ | NM_020663 | 3.259276 |
| ACAA2 | NM_006111 | 3.259182 |
| CLIC4 | NM_013943 | 3.258564 |
| BTN1A1 | NM_001732 | 3.257804 |
| NEK3 | NM_002498 | 3.256211 |
| USP6NL | BC010351 | 3.255466 |
| GLCE | NM_015554 | 3.254811 |
| C15orf23 | BC004543 | 3.254667 |
| IL16 | NM_172217 | 3.253755 |
| WFDC1 | NM_021197 | 3.253125 |
| BEX2 | NM_032621 | 3.252596 |
| FLJ22374 | NM_032222 | 3.252083 |
| OAS3 | NM_006187 | 3.251476 |
| MEIS3P1 | NR_002211 | 3.249242 |
| PDZRN3 | NM_015009 | 3.246561 |
| RUNX2 | NM_004348 | 3.24649 |
| DBN1 | NM_080881 | 3.246269 |
| DET1 | NM_017996 | 3.245294 |
| KIF5C | AB011103 | 3.244865 |
| GNB5 | BC011671 | 3.244785 |
| LRRC44 | NM_145258 | 3.241809 |
| C20orf23 | NM_024704 | 3.239334 |
| BTBD12 | NM_032444 | 3.239054 |
| TMCC2 | NM_014858 | 3.238381 |
| KCTD9 | AF130091 | 3.235995 |
| GNAZ | NM_002073 | 3.23433 |
| OR7D2 | NM_175883 | 3.233977 |
| INCA | NM_001007232 | 3.233731 |
| CLSPN | NM_022111 | 3.23358 |
| MGC29891 | NM_144618 | 3.233248 |
| SLC38A1 | NM_030674 | 3.232994 |
| MTAP | NM_002451 | 3.231367 |
| ESR1 | NM_000125 | 3.230578 |
| SYTL2 | NM_206927 | 3.228135 |
| VPS29 | NM_057180 | 3.227869 |
| SENP6 | NM_015571 | 3.224629 |
| FLJ31196 | NM_152908 | 3.22441 |
| SSR3 | NM_007107 | 3.224254 |
| HERC6 | NM_001013002 | 3.222268 |
| CGI-115 | NM_016052 | 3.221818 |
| FLJ23861 | NM_152519 | 3.217221 |
| RAD51AP1 | NM_006479 | 3.216798 |
| KIAA1706 | NM_030636 | 3.214926 |
| CPS1 | NM_001875 | 3.214132 |
| CAMK2N1 | AF116637 | 3.213493 |
| VANGL1 | NM_138959 | 3.211247 |
| SLITRK4 | NM_173078 | 3.211099 |
| TTTY14 | NR_001543 | 3.210541 |
| FILIP1 | NM_015687 | 3.210301 |
| ULBP1 | NM_025218 | 3.209556 |
| KIAA1598 | NM_018330 | 3.207887 |
| SEL1L | NM_005065 | 3.206849 |
| GPX7 | NM_015696 | 3.206106 |
| FRY | NM_023037 | 3.205088 |
| FAM54A | NM_138419 | 3.203536 |
| NDUFC2 | NM_004549 | 3.202849 |
| C10orf33 | NM_032709 | 3.201369 |
| HLA-DQB1 | M20432 | 3.200509 |
| SEPX1 | NM_016332 | 3.198223 |
| CTSZ | NM_001336 | 3.197035 |
| LPHN3 | NM_015236 | 3.196392 |
| TTLL1 | NM_001008572 | 3.195292 |
| FAM29A | NM_017645 | 3.194484 |
| C1orf103 | NM_018372 | 3.194064 |
| CCDC11 | NM_145020 | 3.193994 |
| VSNL1 | NM_003385 | 3.192879 |
| AHR | NM_001621 | 3.192559 |
| TPM2 | NM_213674 | 3.19214 |
| C21orf129 | NM_152506 | 3.191941 |
| ARHGAP24 | NM_031305 | 3.189294 |
| HLF | NM_002126 | 3.188919 |
| CFLAR | NM_003879 | 3.187055 |
| WDR63 | NM_145172 | 3.186415 |
| FLJ14981 | AK027887 | 3.185397 |
| RIPK3 | NM_006871 | 3.182768 |
| IARS | NM_013417 | 3.18229 |
| NCF2 | NM_000433 | 3.177851 |
| TRIP13 | NM_004237 | 3.176354 |
| PSG9 | NM_002784 | 3.175228 |
| PRO2964 | AF119910 | 3.175142 |
| C6orf167 | CR749603 | 3.174191 |
| POLH | NM_006502 | 3.173187 |
| SORT1 | NM_002959 | 3.172829 |
| THEM4 | NM_176853 | 3.172209 |
| ZNF302 | NM_018443 | 3.171911 |
| SEMA3B | NM_004636 | 3.171459 |
| CCL16 | NM_004590 | 3.171303 |
| TTC7B | NM_001010854 | 3.170354 |
| ATXN3 | NM_004993 | 3.170005 |
| ARMC9 | NM_025139 | 3.167385 |
| RAB28 | NM_004249 | 3.166125 |
| OXCT1 | NM_000436 | 3.160374 |
| NIT2 | NM_020202 | 3.158876 |
| TMEM50A | NM_014313 | 3.158731 |
| IL11RA | NM_004512 | 3.158614 |
| RAB9B | NM_016370 | 3.158507 |
| GGH | NM_003878 | 3.15709 |
| RAD18 | NM_020165 | 3.156954 |
| STOML1 | NM_004809 | 3.156855 |
| HNMT | NM_006895 | 3.155921 |
| MLF1IP | NM_024629 | 3.155765 |
| DKFZP564J102 | NM_015398 | 3.155688 |
| USP40 | NM_018218 | 3.155369 |
| FLJ39822 | NM_173512 | 3.154614 |
| NCALD | NM_032041 | 3.149628 |
| PSME3 | NM_005789 | 3.149549 |
| STX6 | AK056657 | 3.147773 |
| DARS2 | NM_018122 | 3.147528 |
| TFCP2L1 | NM_014553 | 3.146471 |
| TNFSF15 | NM_005118 | 3.146394 |
| C6orf61 | AK000177 | 3.14632 |
| ZWINTAS | X98261 | 3.145227 |
| WDFY3 | NM_014991 | 3.144727 |
| PRTFDC1 | NM_020200 | 3.143171 |
| TTC18 | NM_145170 | 3.140614 |
| TUBB6 | NM_032525 | 3.140236 |
| DENND2A | NM_015689 | 3.136759 |
| FMO4 | NM_002022 | 3.133596 |
| PLEKHA6 | NM_014935 | 3.132926 |
| CAMTA1 | NM_015215 | 3.132279 |
| GATM | NM_001482 | 3.132139 |
| TSPAN18 | NM_130783 | 3.131818 |
| MAP1LC3C | NM_001004343 | 3.130403 |
| NEK11 | NM_024800 | 3.130193 |
| EYA1 | NM_000503 | 3.128251 |
| FLJ37035 | AK094354 | 3.126509 |
| MGC11332 | NM_032718 | 3.125543 |
| GAS2L3 | BX649059 | 3.124116 |
| TPM4 | NM_003290 | 3.123718 |
| APOD | NM_001647 | 3.123582 |
| SLC25A24 | NM_213651 | 3.121966 |
| FAM90A1 | NM_018088 | 3.120527 |
| KIAA1660 | AB051447 | 3.12005 |
| ABLIM1 | NM_001003408 | 3.118527 |
| C14orf106 | NM_018353 | 3.117284 |
| THTPA | NM_024328 | 3.117212 |
| SMARCAL1 | NM_014140 | 3.116966 |
| ASCC3 | NM_022091 | 3.113601 |
| LARGE | NM_004737 | 3.113346 |
| SPFH2 | NM_001003790 | 3.112167 |
| GOPC | NM_020399 | 3.112024 |
| KIT | NM_000222 | 3.111657 |
| DCN | NM_001920 | 3.110332 |
| AQP11 | NM_173039 | 3.108382 |
| CNTNAP4 | AK054786 | 3.105763 |
| PPIC | NM_000943 | 3.105072 |
| MET | NM_000245 | 3.103874 |
| TRIM9 | NM_052978 | 3.10349 |
| CALCRL | NM_005795 | 3.102905 |
| KIAA0323 | NM_015299 | 3.101334 |
| NUCKS1 | NM_022731 | 3.099878 |
| CTTN | NM_005231 | 3.09825 |
| ARHGEF5 | NM_005435 | 3.095038 |
| PSD3 | NM_015310 | 3.094801 |
| HN1 | NM_001002033 | 3.092488 |
| C12orf26 | NM_032230 | 3.088863 |
| DIAPH2 | NM_006729 | 3.087686 |
| CCDC36 | NM_178173 | 3.085518 |
| MUM1 | NM_032853 | 3.083887 |
| C20orf161 | NM_033421 | 3.079978 |
| CSPG2 | NM_004385 | 3.078553 |
| GYS1 | NM_002103 | 3.078125 |
| DNMT3B | NM_175850 | 3.077854 |
| RUFY2 | NM_017987 | 3.077852 |
| CCDC56 | NM_001040431 | 3.076788 |
| MGP | NM_000900 | 3.076591 |
| FARSLB | AK001025 | 3.076533 |
| RALGPS2 | NM_152663 | 3.076139 |
| ZDHHC3 | NM_016598 | 3.074463 |
| GRK4 | NM_001004057 | 3.072186 |
| ZDHHC2 | NM_016353 | 3.071559 |
| ITGA7 | NM_002206 | 3.063026 |
| AGTPBP1 | NM_015239 | 3.062117 |
| SSTR1 | NM_001049 | 3.061342 |
| ZNF616 | BC032805 | 3.058811 |
| MASP1 | NM_139125 | 3.057712 |
| LACTB2 | NM_016027 | 3.055208 |
| FLJ25791 | NM_173559 | 3.055012 |
| DHCR24 | NM_014762 | 3.052428 |
| FLJ37453 | AK094772 | 3.052048 |
| TSPAN13 | NM_014399 | 3.049358 |
| C18orf50 | AF363068 | 3.048607 |
| APP | NM_000484 | 3.046595 |
| ZFHX2 | NM_033400 | 3.04602 |
| C14orf159 | NM_024952 | 3.045653 |
| MX1 | NM_002462 | 3.045232 |
| MLCK | NM_182493 | 3.045068 |
| FLJ33630 | AF088062 | 3.044142 |
| PIGK | NM_005482 | 3.043712 |
| DYNLT3 | NM_006520 | 3.042209 |
| CPVL | NM_031311 | 3.040734 |
| MRPL30 | NM_145212 | 3.040382 |
| KCNS3 | NM_002252 | 3.040225 |
| KIAA1794 | NM_018193 | 3.039143 |
| HFE | NM_139004 | 3.03912 |
| LIMD2 | NM_030576 | 3.038459 |
| CBWD2 | NM_172003 | 3.038356 |
| C11orf52 | NM_080659 | 3.038041 |
| CDC42EP2 | NM_006779 | 3.037798 |
| VEGFC | NM_005429 | 3.037082 |
| RAD54B | NM_012415 | 3.036534 |
| MTMR2 | NM_201278 | 3.036461 |
| NCOA6 | NM_014071 | 3.033391 |
| TRAF3 | NM_145725 | 3.032847 |
| TRIM32 | NM_012210 | 3.030411 |
| STOX2 | NM_020225 | 3.030045 |
| PHKB | NM_000293 | 3.029776 |
| ATG10 | NM_031482 | 3.028227 |
| TMSB4X | NM_021109 | 3.024552 |
| COL2A1 | NM_001844 | 3.024115 |
| WDR1 | NM_017491 | 3.024044 |
| EPB41L4A | NM_022140 | 3.022432 |
| EDG8 | NM_030760 | 3.020299 |
| DGKH | BC044822 | 3.019944 |
| ACTC | NM_005159 | 3.018341 |
| LIG4 | NM_002312 | 3.017998 |
| C8orf40 | NM_138436 | 3.017849 |
| DLD | NM_000108 | 3.016981 |
| WDR42A | BC111063 | 3.016241 |
| RSPO2 | NM_178565 | 3.014377 |
| SGPL1 | NM_003901 | 3.012479 |
| HIST1H4B | NM_003544 | 3.010863 |
| TK1 | NM_003258 | 3.010833 |
| CDKN2B | NM_078487 | 3.010561 |
| NPAL3 | NM_020448 | 3.009585 |
| TSHZ3 | NM_020856 | 3.009415 |
| ARVCF | NM_001670 | 3.009379 |
| GARS | NM_002047 | 3.008617 |
| HIBCH | NM_014362 | 3.008234 |
| TBC1D23 | NM_018309 | 3.008033 |
| CDC42EP4 | NM_012121 | 3.008022 |
| SRP19 | NM_003135 | 3.00797 |
| BBS1 | NM_024649 | 3.005547 |
| DRCTNNB1A | NM_032581 | 3.004651 |
| BTN2A1 | NM_078476 | 3.004615 |
| ATP1A2 | NM_000702 | 3.004548 |
| NEK9 | NM_033116 | 3.004528 |
| DNAJA5 | NM_194283 | 3.004113 |
| E2F8 | NM_024680 | 3.004099 |
| DFNB59 | BC020859 | 3.00283 |
| KIF18A | NM_031217 | 3.002004 |
| C4orf15 | NM_024511 | 3.001701 |
| GNPTAB | NM_024312 | 3.000304 |
| FLJ13231 | NM_023073 | 3.000244 |
| C11orf51 | NM_014042 | 2.999127 |
| ST3GAL6 | NM_006100 | 2.998656 |
| THAP2 | NM_031435 | 2.998419 |
| RPS18 | NM_022551 | 2.997884 |
| THBS4 | NM_003248 | 2.997793 |
| HERC4 | AY650034 | 2.995694 |
| ASH1L | NM_018489 | 2.99527 |
| TFPI | NM_006287 | 2.994957 |
| R3HDM1 | NM_015361 | 2.994463 |
| C14orf113 | AK000041 | 2.99293 |
| CLCA3 | NM_004921 | 2.99198 |
| GAD1 | NM_000817 | 2.988541 |
| NFYB | NM_006166 | 2.987963 |
| RUNX1 | NM_001001890 | 2.98771 |
| HHLA3 | NM_007071 | 2.987597 |
| TRIM2 | NM_015271 | 2.987301 |
| ASTN2 | NM_198188 | 2.985474 |
| CFL2 | NM_021914 | 2.983879 |
| HNRPA3 | NM_194247 | 2.981788 |
| OR7E156P | NR_002171 | 2.980099 |
| NEDD8 | AK125214 | 2.979635 |
| FIGNL1 | NM_022116 | 2.977639 |
| HBS1L | NM_006620 | 2.977608 |
| COLEC12 | NM_030781 | 2.976161 |
| HMCN1 | NM_031935 | 2.974325 |
| XG | NM_175569 | 2.972521 |
| NR4A3 | NM_173199 | 2.970082 |
| FBXO15 | NM_152676 | 2.969167 |
| TAGLN | NM_001001522 | 2.968132 |
| PRR7 | NM_030567 | 2.96729 |
| MGAT2 | NM_002408 | 2.966952 |
| ZNF396 | NM_145756 | 2.96587 |
| MKS1 | NM_017777 | 2.965237 |
| SFRS6 | NM_006275 | 2.963794 |
| COLQ | NM_080542 | 2.96362 |
| GRB10 | NM_001001555 | 2.962349 |
| MAGED4 | NM_030801 | 2.962333 |
| PLIN | NM_002666 | 2.960788 |
| RFPL1S | NR_002727 | 2.958135 |
| APOLD1 | NM_030817 | 2.957344 |
| SRI | NM_003130 | 2.956947 |
| CECR1 | NM_017424 | 2.956735 |
| PKMYT1 | NM_004203 | 2.95635 |
| RPS12 | NM_001016 | 2.953502 |
| CCL28 | NM_148672 | 2.953308 |
| RPE | NM_006916 | 2.951212 |
| HERC3 | NM_014606 | 2.950166 |
| HKDC1 | NM_025130 | 2.946146 |
| ACTR2 | NM_001005386 | 2.946036 |
| tcag7.1239 | XM_926769 | 2.946006 |
| XKR6 | NM_173683 | 2.945808 |
| C15orf20 | NM_025049 | 2.944386 |
| FLJ40330 | BC031698 | 2.94254 |
| ZWILCH | NM_017975 | 2.940563 |
| SEMA7A | NM_003612 | 2.94054 |
| C1QTNF2 | NM_031908 | 2.94046 |
| COX11 | NM_004375 | 2.939368 |
| KIAA0367 | NM_015225 | 2.938988 |
| C12orf42 | NM_198521 | 2.938502 |
| RPL34 | NM_033625 | 2.938393 |
| HMMR | NM_012484 | 2.938158 |
| FBLN2 | NM_001004019 | 2.93648 |
| CCL8 | NM_005623 | 2.935882 |
| PARD3 | AF196185 | 2.935761 |
| ANKRD6 | AB023174 | 2.935363 |
| MGC40405 | NM_152789 | 2.935057 |
| C20orf141 | AK129653 | 2.934861 |
| C14orf145 | BC036939 | 2.934136 |
| ENPEP | NM_001977 | 2.934057 |
| SCUBE3 | NM_152753 | 2.933921 |
| H2AFJ | NM_018267 | 2.931971 |
| SERPINB13 | NM_012397 | 2.931197 |
| TMSL8 | NM_021992 | 2.929144 |
| C1orf41 | NM_016126 | 2.92875 |
| PPP1R12B | NM_032103 | 2.927801 |
| SFRP4 | NM_003014 | 2.927766 |
| FER1L3 | NM_013451 | 2.924057 |
| HARS2 | AK074304 | 2.921886 |
| DNAH11 | NM_003777 | 2.921319 |
| C3orf63 | NM_015224 | 2.920282 |
| TMEM102 | NM_178518 | 2.920051 |
| C16orf55 | NM_153025 | 2.918954 |
| MGC72104 | NM_207350 | 2.918906 |
| LRFN3 | NM_024509 | 2.918657 |
| SDSL | NM_138432 | 2.917115 |
| STAMBPL1 | NM_020799 | 2.916967 |
| C14orf149 | NM_144581 | 2.916871 |
| TPST1 | NM_003596 | 2.915658 |
| ALDH2 | NM_000690 | 2.915252 |
| PTPLAD2 | NM_001010915 | 2.913225 |
| SURF2 | NM_017503 | 2.912423 |
| SYNPO2L | NM_024875 | 2.911383 |
| FMO3 | NM_001002294 | 2.908101 |
| ADAMTS5 | NM_007038 | 2.907855 |
| ACVR1C | NM_145259 | 2.907293 |
| PRKAG3 | NM_017431 | 2.907237 |
| RCCD1 | NM_033544 | 2.906926 |
| OR5I1 | NM_006637 | 2.906798 |
| FLJ20160 | NM_017694 | 2.904803 |
| SGTB | NM_019072 | 2.904787 |
| FGFR1OP | NM_007045 | 2.904464 |
| SEC31L1 | AK128047 | 2.904069 |
| NAP1L2 | NM_021963 | 2.903796 |
| CEP290 | NM_025114 | 2.903364 |
| RPS23 | NM_001025 | 2.903185 |
| HISPPD2A | NM_014659 | 2.902931 |
| NHLH2 | NM_005599 | 2.90235 |
| FLJ40243 | NM_173489 | 2.901563 |
| ARID5B | NM_032199 | 2.901151 |
| TRAF1 | NM_005658 | 2.899495 |
| TACC3 | NM_006342 | 2.899436 |
| C1orf151 | NM_001032363 | 2.898875 |
| PLAU | NM_002658 | 2.898808 |
| CRI1 | NM_014335 | 2.897865 |
| MTMR4 | NM_004687 | 2.892802 |
| MYOCD | NM_153604 | 2.891186 |
| GLYATL2 | NM_145016 | 2.89074 |
| C6orf157 | NM_198920 | 2.889354 |
| C11orf54 | NM_014039 | 2.888903 |
| GBA3 | NM_020973 | 2.885199 |
| KIAA0157 | NM_032182 | 2.884935 |
| CST6 | NM_001323 | 2.884909 |
| HAS3 | NM_138612 | 2.883679 |
| P4HB | AB062434 | 2.882643 |
| NLN | NM_020726 | 2.881284 |
| ARG2 | NM_001172 | 2.88109 |
| ALS2CR2 | AF116618 | 2.880911 |
| CACNA1C | NM_000719 | 2.879649 |
| FLJ39609 | AK096928 | 2.879549 |
| DCBLD1 | NM_173674 | 2.878844 |
| TINAGL1 | NM_022164 | 2.877605 |
| NNMT | NM_006169 | 2.877462 |
| HSPA4L | NM_014278 | 2.876563 |
| FKBP1A | NM_000801 | 2.874222 |
| TYMS | NM_001071 | 2.873594 |
| SUV420H1 | NM_017635 | 2.872886 |
| HS3ST2 | NM_006043 | 2.87228 |
| C10orf46 | NM_153810 | 2.871848 |
| CALM1 | NM_006888 | 2.869625 |
| ZNF273 | BC008386 | 2.868227 |
| EFEMP1 | NM_004105 | 2.867268 |
| CYB5R2 | NM_016229 | 2.864822 |
| C1RL | NM_016546 | 2.864414 |
| ATPBD4 | NM_080650 | 2.864412 |
| LAMA4 | NM_002290 | 2.863195 |
| RPLP2 | NM_001004 | 2.862476 |
| SLITRK5 | NM_015567 | 2.862273 |
| ROR1 | NM_005012 | 2.861342 |
| RABL4 | NM_006860 | 2.861067 |
| C14orf156 | NM_031210 | 2.861043 |
| HIP1 | NM_005338 | 2.858361 |
| MGC16824 | NM_020314 | 2.857542 |
| XPO6 | NM_015171 | 2.854681 |
| C6orf203 | NM_016487 | 2.854206 |
| KIAA1394 | BC036557 | 2.853017 |
| ZSWIM5 | AB040944 | 2.851849 |
| C12orf24 | NM_013300 | 2.851758 |
| ID4 | NM_001546 | 2.851691 |
| STMN3 | NM_015894 | 2.850758 |
| RPL22 | AF136171 | 2.850459 |
| DCTN6 | NM_006571 | 2.849692 |
| PALLD | NM_016081 | 2.848328 |
| DCD | NM_053283 | 2.848229 |
| PTPN18 | NM_014369 | 2.847595 |
| SMYD2 | NM_020197 | 2.846814 |
| DKFZP586K1520 | AL050153 | 2.845582 |
| PPA2 | NM_176869 | 2.844719 |
| RASAL2 | NM_170692 | 2.844098 |
| tcag7.981 | NM_175061 | 2.84398 |
| K-ALPHA-1 | NM_006082 | 2.843305 |
| CXorf12 | NM_003492 | 2.842412 |
| LYPLAL1 | NM_138794 | 2.841706 |
| CROP | NM_006107 | 2.841391 |
| ARHGEF17 | NM_014786 | 2.840594 |
| HEATR3 | NM_182922 | 2.839989 |
| PKN2 | AF118089 | 2.839778 |
| FHL2 | NM_201555 | 2.839522 |
| CCND2 | NM_001759 | 2.838282 |
| C17orf67 | BC041467 | 2.838 |
| NAV1 | NM_020443 | 2.837199 |
| C11orf67 | NM_024684 | 2.836689 |
| CD28 | NM_006139 | 2.836332 |
| C16orf74 | BC009078 | 2.835911 |
| NDUFB2 | NM_004546 | 2.835826 |
| EFHC1 | AY608689 | 2.835484 |
| KIAA0738 | NM_014719 | 2.83438 |
| FLJ39378 | AK097550 | 2.833744 |
| C11orf1 | NM_022761 | 2.833511 |
| ST13 | NM_003932 | 2.831367 |
| CYB5R4 | NM_016230 | 2.830733 |
| CLIC3 | NM_004669 | 2.829972 |
| CKAP1 | NM_001281 | 2.829962 |
| KIAA1727 | NM_033393 | 2.829071 |
| EHD3 | NM_014600 | 2.828919 |
| NACAP1 | NR_002182 | 2.828444 |
| NP1166121 | NP1166121 | 2.827777 |
| NMNAT1 | NM_022787 | 2.827487 |
| PDLIM7 | NM_005451 | 2.827043 |
| ZNF702 | AK023047 | 2.826792 |
| ETV1 | NM_004956 | 2.826577 |
| PDE3A | NM_000921 | 2.826007 |
| C1orf167 | AL834308 | 2.825247 |
| TRSPAP1 | NM_017846 | 2.824447 |
| GLDC | NM_000170 | 2.821985 |
| C3orf60 | NM_199417 | 2.821629 |
| RASGRP1 | NM_005739 | 2.820404 |
| BTBD14B | NM_052876 | 2.820186 |
| EXOC6 | NM_001013848 | 2.820045 |
| IGFBP2 | NM_000597 | 2.81968 |
| IL23R | NM_144701 | 2.819613 |
| DOCK1 | NM_001380 | 2.819375 |
| MYOC | NM_000261 | 2.818993 |
| RBMS1 | NM_002897 | 2.818429 |
| ANKRD11 | NM_013275 | 2.817978 |
| ZNF527 | AK091585 | 2.817689 |
| PHOSPHO2 | NM_001008489 | 2.817373 |
| FTH1 | NM_002032 | 2.81415 |
| DSC3 | NM_024423 | 2.813387 |
| PFDN1 | NM_002622 | 2.812863 |
| OAS1 | NM_002534 | 2.812412 |
| RP1-32F7.2 | NM_173698 | 2.810415 |
| FAT | NM_005245 | 2.809255 |
| CILP | NM_003613 | 2.805947 |
| SLC35E3 | NM_018656 | 2.805874 |
| TRIO | AK131423 | 2.805045 |
| SLC27A6 | NM_001017372 | 2.80503 |
| RTN2 | NM_005619 | 2.803646 |
| KCNJ2 | NM_000891 | 2.801604 |
| TRIM14 | NM_014788 | 2.800928 |
| CBR4 | NM_032783 | 2.800709 |
| FLJ32679 | NM_001012452 | 2.799631 |
| FLJ10357 | NM_018071 | 2.799373 |
| COQ2 | NM_015697 | 2.798793 |
| ZNF278 | NM_032051 | 2.797908 |
| C3orf9 | NM_152305 | 2.797678 |
| IGSF10 | NM_178822 | 2.797669 |
| COL4A5 | NM_033380 | 2.795885 |
| ZADH1 | NM_152444 | 2.795097 |
| TBC1D9B | NM_198868 | 2.794955 |
| FLJ40869 | NM_182625 | 2.793933 |
| LHFP | NM_005780 | 2.793338 |
| RPL21 | NM_000982 | 2.792757 |
| C21orf58 | AY039244 | 2.791261 |
| MTERF | NM_006980 | 2.790466 |
| CYP20A1 | NM_177538 | 2.787118 |
| JARID2 | NM_004973 | 2.786306 |
| PHF20 | AK090798 | 2.786154 |
| FAM120AOS | NM_198841 | 2.784989 |
| JAK2 | NM_004972 | 2.784452 |
| C14orf125 | AL117511 | 2.783489 |
| DLL1 | NM_005618 | 2.782006 |
| PSG4 | NM_213633 | 2.781666 |
| KIF3C | NM_002254 | 2.781343 |
| SLC35B4 | NM_032826 | 2.779356 |
| MAPKAP1 | NM_001006617 | 2.777335 |
| HOMER1 | NM_004272 | 2.775265 |
| FAM59A | NM_022751 | 2.775118 |
| C7 | NM_000587 | 2.774295 |
| ATOH8 | NM_032827 | 2.774251 |
| CSN3 | NM_005212 | 2.77387 |
| ADAMTS3 | NM_014243 | 2.773824 |
| KCND2 | NM_012281 | 2.773275 |
| IGF1R | NM_000875 | 2.772587 |
| PDHB | NM_000925 | 2.770695 |
| QDPR | NM_000320 | 2.769775 |
| INHBA | NM_002192 | 2.769615 |
| ATF7 | BC042363 | 2.766985 |
| CKMT2 | NM_001825 | 2.766687 |
| KIAA1430 | AK024878 | 2.766261 |
| CYP3A5 | NM_000777 | 2.765881 |
| STC2 | NM_003714 | 2.764344 |
| ARV1 | NM_022786 | 2.761891 |
| PSG3 | NM_021016 | 2.761182 |
| MGC20470 | NM_145053 | 2.760395 |
| GNA14 | NM_004297 | 2.759932 |
| CHMP2B | NM_014043 | 2.75993 |
| NUAK1 | NM_014840 | 2.758899 |
| KRTAP3-3 | NM_033185 | 2.75807 |
| CAPS | NM_004058 | 2.756119 |
| FANCL | NM_018062 | 2.7557 |
| ST18 | NM_014682 | 2.755662 |
| IL15 | NM_172174 | 2.753805 |
| KIDINS220 | NM_020738 | 2.753296 |
| WSB1 | NM_015626 | 2.75306 |
| PITPNM2 | NM_020845 | 2.753034 |
| NFE2L3 | NM_004289 | 2.750516 |
| ANKRD13A | NM_033121 | 2.749753 |
| IL13RA2 | NM_000640 | 2.749572 |
| DKFZP761M1511 | AK096661 | 2.749377 |
| WDR77 | NM_024102 | 2.749004 |
| SIRT3 | NM_012239 | 2.7468 |
| FLJ36031 | NM_175884 | 2.745388 |
| ACYP2 | NM_138448 | 2.744914 |
| GCLC | M90656 | 2.74489 |
| ELF2 | NM_201999 | 2.74343 |
| UNC5B | NM_170744 | 2.740649 |
| ANTXR1 | NM_032208 | 2.740308 |
| TRIM6 | NM_001003818 | 2.738393 |
| WDR41 | NM_018268 | 2.738038 |
| C9orf94 | NM_001040272 | 2.737061 |
| ANXA2P1 | NR_001562 | 2.736611 |
| C16orf35 | NM_001039476 | 2.736585 |
| TRPM7 | NM_017672 | 2.736017 |
| OSMR | NM_003999 | 2.734569 |
| ANKRD20A2 | NM_001012421 | 2.734372 |
| PZP | NM_002864 | 2.734178 |
| CCDC76 | NM_019083 | 2.733943 |
| RPL23A | NM_000984 | 2.733647 |
| SNRPG | NM_003096 | 2.733564 |
| GPR19 | NM_006143 | 2.731889 |
| TMC7 | NM_024847 | 2.731704 |
| CDH15 | NM_004933 | 2.731474 |
| MGC40499 | NM_152755 | 2.731431 |
| SPTY2D1 | NM_194285 | 2.72944 |
| DLGAP4 | NM_014902 | 2.728678 |
| PDLIM5 | NM_006457 | 2.72716 |
| C14orf131 | NM_018335 | 2.726912 |
| SYT11 | NM_152280 | 2.725458 |
| RPS6KA6 | NM_014496 | 2.725375 |
| SEC15L2 | AK023791 | 2.725236 |
| C6 | NM_000065 | 2.724595 |
| CDADC1 | NM_030911 | 2.723729 |
| ARMCX3 | NM_016607 | 2.723551 |
| MORN2 | NM_194270 | 2.722854 |
| TSPYL4 | NM_021648 | 2.721144 |
| GMIP | NM_016573 | 2.72107 |
| FLJ40852 | NM_173677 | 2.720762 |
| NFIB | NM_005596 | 2.719973 |
| C6orf125 | NM_032340 | 2.718385 |
| SLC31A2 | NM_001860 | 2.717981 |
| ANKRD19 | NM_001010925 | 2.716159 |
| MAPK12 | NM_002969 | 2.716063 |
| TncRNA | NR_002802 | 2.715975 |
| ZNF31 | NM_145238 | 2.715229 |
| MRPS10 | NM_018141 | 2.715186 |
| PCDHB6 | NM_018939 | 2.714095 |
| DDX17 | NM_006386 | 2.713904 |
| RRAS2 | NM_012250 | 2.713746 |
| COMMD8 | NM_017845 | 2.712867 |
| C1orf165 | NM_024603 | 2.712833 |
| OR51E2 | NM_030774 | 2.712716 |
| FGFR1 | NM_023110 | 2.712391 |
| CCDC62 | NM_032573 | 2.712013 |
| LSM8 | NM_016200 | 2.711782 |
| ZCCHC7 | NM_032226 | 2.710855 |
| MGC21675 | NM_052861 | 2.709972 |
| TMEM155 | NM_152399 | 2.708516 |
| ORAOV1 | NM_153451 | 2.705842 |
| CEP72 | NM_018140 | 2.70568 |
| GFOD1 | NM_018988 | 2.70557 |
| PHGDH | NM_006623 | 2.705506 |
| CCDC74B | NM_207310 | 2.70474 |
| TMTC2 | NM_152588 | 2.703412 |
| C1orf118 | NM_001039463 | 2.703054 |
| GHR | NM_000163 | 2.701613 |
| NDUFA6 | NM_002490 | 2.701331 |
| STK10 | NM_005990 | 2.699381 |
| DNAJC18 | NM_152686 | 2.697695 |
| WNT5B | NM_030775 | 2.694306 |
| RAB33B | NM_031296 | 2.692848 |
| ZNF519 | NM_145287 | 2.692638 |
| ETV5 | NM_004454 | 2.692224 |
| BTBD4 | AK126720 | 2.691836 |
| UEVLD | NM_018314 | 2.689681 |
| TCTA | NM_022171 | 2.68965 |
| FOLH1 | NM_004476 | 2.689575 |
| HNRPU | NM_031844 | 2.68858 |
| ZNF650 | NM_172070 | 2.687917 |
| CTGLF1 | NM_133446 | 2.687783 |
| HIST1H4E | NM_003545 | 2.687463 |
| LILRA3 | NM_006865 | 2.686159 |
| ACCN5 | NM_017419 | 2.685002 |
| S100A4 | NM_002961 | 2.684199 |
| MGC26356 | NM_001039758 | 2.68374 |
| SEC24D | AK000709 | 2.68189 |
| CRI2 | NM_153232 | 2.681665 |
| COL10A1 | NM_000493 | 2.681645 |
| DCK | NM_000788 | 2.681574 |
| KCTD10 | NM_031954 | 2.681374 |
| TSGA2 | NM_080860 | 2.681253 |
| QKI | NM_006775 | 2.681059 |
| C3orf64 | NM_173654 | 2.680721 |
| SERINC2 | NM_178865 | 2.678583 |
| MGC23909 | NM_174909 | 2.677651 |
| CCBE1 | NM_133459 | 2.677431 |
| RPS27A | NM_002954 | 2.676563 |
| TAOK3 | NM_016281 | 2.67481 |
| MON2 | NM_015026 | 2.674103 |
| NGFRAP1 | NM_014380 | 2.674015 |
| DBNL | NM_014063 | 2.673629 |
| CRNKL1 | NM_016652 | 2.672995 |
| ERN1 | NM_001433 | 2.67294 |
| DHX40 | NM_024612 | 2.671174 |
| PPHLN1 | NM_201515 | 2.670841 |
| C6orf85 | BC022217 | 2.67022 |
| SESN3 | NM_144665 | 2.669803 |
| TCF4 | NM_003199 | 2.667698 |
| CXADR | NM_001338 | 2.66764 |
| KITLG | NM_000899 | 2.662568 |
| VPS41 | BX648347 | 2.66226 |
| HTR2B | NM_000867 | 2.662167 |
| ITGAV | NM_002210 | 2.662098 |
| AGA | NM_000027 | 2.662037 |
| C10orf22 | NM_032804 | 2.661064 |
| C14orf108 | NM_018229 | 2.660643 |
| C14orf128 | BC007251 | 2.659789 |
| MDS025 | NM_021825 | 2.659725 |
| OXTR | NM_000916 | 2.65853 |
| STAT1 | NM_007315 | 2.657644 |
| LRRK2 | NM_198578 | 2.657344 |
| TPM3 | NM_152263 | 2.657267 |
| SERPINF1 | NM_002615 | 2.657173 |
| HERC2 | NM_004667 | 2.657009 |
| C15orf5 | BC069765 | 2.656785 |
| SLC45A4 | BC033223 | 2.656326 |
| GINS1 | NM_021067 | 2.655347 |
| C16orf75 | NM_152308 | 2.654981 |
| ODF2L | NM_020729 | 2.654791 |
| MTRF1 | NM_004294 | 2.653855 |
| PRO0471 | AF111846 | 2.653595 |
| KIAA0484 | AB007953 | 2.653484 |
| ATP11A | NM_015205 | 2.651828 |
| TNIP3 | NM_024873 | 2.651406 |
| KIAA0586 | NM_014749 | 2.650275 |
| TCP1 | NM_030752 | 2.649725 |
| CYP39A1 | NM_016593 | 2.647266 |
| CDRT1 | NM_006382 | 2.646879 |
| TFAM | NM_003201 | 2.645534 |
| TUBB3 | NM_006086 | 2.644356 |
| KLHL20 | BC005253 | 2.644178 |
| LAMB1 | NM_002291 | 2.644144 |
| IFNE1 | NM_176891 | 2.644065 |
| ZNF545 | AB075828 | 2.643999 |
| PLEKHA5 | NM_019012 | 2.64356 |
| ZNF354A | NM_005649 | 2.642192 |
| HBM | NM_001003938 | 2.641714 |
| TNFRSF10C | NM_003841 | 2.641226 |
| CLK4 | NM_020666 | 2.640935 |
| RTN4RL1 | NM_178568 | 2.640814 |
| PPP4R1 | NM_005134 | 2.639392 |
| DNAJB6 | NM_005494 | 2.639046 |
| ANKRD25 | NM_015493 | 2.638494 |
| RPS10 | NM_001014 | 2.637325 |
| ZNF606 | NM_025027 | 2.636428 |
| C13orf1 | NM_020456 | 2.636167 |
| SPP1 | NM_000582 | 2.635687 |
| FCMD | NM_006731 | 2.635083 |
| CRYBB2 | NM_000496 | 2.633741 |
| CPT1C | NM_152359 | 2.632333 |
| KCNE3 | NM_005472 | 2.632323 |
| KBTBD8 | NM_032505 | 2.631815 |
| KCNN4 | NM_002250 | 2.631306 |
| EPR1 | NR_002219 | 2.629043 |
| PHYHD1 | NM_174933 | 2.62852 |
| SERPINB8 | NM_198833 | 2.628398 |
| SLC14A2 | AK074236 | 2.627969 |
| DGKZ | NM_003646 | 2.627319 |
| AUTS2 | NM_015570 | 2.627299 |
| C21orf34 | NM_001005733 | 2.627228 |
| NUDCD1 | NM_032869 | 2.62707 |
| ZCCHC4 | AY629351 | 2.626622 |
| PCSK2 | NM_002594 | 2.624357 |
| RBKS | NM_022128 | 2.623773 |
| ARMC2 | NM_032131 | 2.623622 |
| FANCF | NM_022725 | 2.623605 |
| FAM86B1 | NM_032916 | 2.62332 |
| GLT8D3 | BC039145 | 2.623284 |
| RORA | NM_134260 | 2.623191 |
| CAST | NM_173060 | 2.623142 |
| BDNF | NM_170735 | 2.623083 |
| CCNJL | NM_024565 | 2.622749 |
| ZBTB8OS | NM_178547 | 2.621913 |
| EPC2 | NM_015630 | 2.621498 |
| DBC1 | NM_014618 | 2.620893 |
| UBE2T | NM_014176 | 2.620497 |
| RPL7 | NM_000971 | 2.620396 |
| ZFYVE16 | NM_014733 | 2.620101 |
| WDR7 | NM_015285 | 2.61972 |
| NPY1R | NM_000909 | 2.619479 |
| ANXA2P3 | NR_001446 | 2.619154 |
| SAMHD1 | NM_015474 | 2.617507 |
| LHX4 | AK096250 | 2.616473 |
| SEMA5B | NM_001031702 | 2.615844 |
| RDH11 | NM_016026 | 2.6143 |
| TMEM100 | NM_018286 | 2.614211 |
| AMOTL2 | NM_016201 | 2.613925 |
| RUNX1T1 | NM_004349 | 2.613814 |
| RPS6KA3 | NM_004586 | 2.61353 |
| CAPN10 | NM_023083 | 2.612291 |
| MGC4562 | NM_133375 | 2.610107 |
| Klkbl4 | AK058068 | 2.609278 |
| DTWD1 | AF168717 | 2.608199 |
| BET1 | NM_005868 | 2.608021 |
| ZNF553 | NM_152652 | 2.608004 |
| DDX50 | NM_024045 | 2.607 |
| C1orf71 | NM_152609 | 2.606424 |
| PDCD6IP | NM_013374 | 2.605939 |
| ZNRF2 | NM_147128 | 2.605721 |
| CHKA | NM_001277 | 2.603241 |
| TMEM140 | AK056910 | 2.602011 |
| JMJD1C | NM_004241 | 2.601735 |
| KRT23 | NM_015515 | 2.600608 |
| FRMD4B | AB023230 | 2.600544 |
| GOLGA3 | NM_005895 | 2.59974 |
| PHF23 | NM_024297 | 2.599691 |
| FHOD3 | NM_025135 | 2.599185 |
| ARHGEF10 | BC026965 | 2.599164 |
| MRPL35 | NM_145644 | 2.5989 |
| KIF23 | NM_138555 | 2.598633 |
| RIC3 | NM_024557 | 2.598515 |
| CSRP2 | NM_001321 | 2.598045 |
| PYCARD | NM_013258 | 2.597728 |
| SERF1B | NM_022978 | 2.596815 |
| GSTA2 | NM_000846 | 2.59626 |
| ZNF546 | NM_178544 | 2.596012 |
| SEMA3C | NM_006379 | 2.594722 |
| SOCS6 | NM_004232 | 2.592285 |
| IQCE | AB028946 | 2.591629 |
| HIST1H4L | NM_003546 | 2.591247 |
| B3GALT4 | NM_003782 | 2.590492 |
| S100A6 | NM_014624 | 2.589637 |
| FLJ36032 | AK093351 | 2.589269 |
| TDRD7 | NM_014290 | 2.588988 |
| GPIAP1 | NM_203364 | 2.588729 |
| PIN4 | NM_006223 | 2.588268 |
| H19 | BC011866 | 2.587689 |
| DUSP19 | NM_080876 | 2.587673 |
| ARL10 | NM_173664 | 2.587656 |
| DDX52 | NM_152300 | 2.585677 |
| ERO1L | NM_014584 | 2.585607 |
| FLJ35348 | NR_002800 | 2.583136 |
| FXN | NM_181425 | 2.583038 |
| VCX2 | NM_016378 | 2.582414 |
| GPR125 | NM_145290 | 2.580809 |
| MALT1 | NM_006785 | 2.580384 |
| JOSD3 | NM_024116 | 2.578969 |
| C15orf29 | NM_024713 | 2.577648 |
| PSMD9 | NM_002813 | 2.576357 |
| SCHIP1 | NM_014575 | 2.575351 |
| OSTM1 | NM_014028 | 2.57462 |
| FLJ38725 | NM_153218 | 2.574217 |
| TERF1 | NM_017489 | 2.573039 |
| TPTE | NM_199261 | 2.572854 |
| HTR3A | NM_213621 | 2.57246 |
| TUBA8 | NM_018943 | 2.572226 |
| BCAT1 | NM_005504 | 2.570311 |
| PRKAB2 | NM_005399 | 2.570129 |
| NUFIP1 | NM_012345 | 2.569904 |
| POLR3H | NM_001018051 | 2.569542 |
| PSG11 | NM_002785 | 2.569172 |
| TMEM107 | NM_032354 | 2.568579 |
| ZNF93 | AK096342 | 2.567765 |
| ANXA10 | NM_007193 | 2.565961 |
| ACADSB | NM_001609 | 2.56484 |
| BVES | NM_147147 | 2.563862 |
| ZNF347 | NM_032584 | 2.563239 |
| C10orf89 | NM_153336 | 2.562844 |
| CAPZA3 | NM_033328 | 2.562435 |
| FLJ30277 | NM_153008 | 2.562192 |
| HSP90AA1 | NM_005348 | 2.562111 |
| TMF1 | NM_007114 | 2.56137 |
| CAPN1 | NM_005186 | 2.560819 |
| LRRC48 | NM_031294 | 2.560238 |
| CTH | NM_001902 | 2.559444 |
| CPA2 | NM_001869 | 2.559194 |
| MAP3K6 | NM_004672 | 2.558378 |
| CREBL2 | NM_001310 | 2.557885 |
| TADA2L | NM_001488 | 2.557456 |
| ADD3 | NM_016824 | 2.557434 |
| LMBRD2 | NM_001007527 | 2.557154 |
| HTRA3 | NM_053044 | 2.55619 |
| AOX1 | NM_001159 | 2.556033 |
| PNPLA4 | NM_004650 | 2.554168 |
| TSKS | NM_021733 | 2.554012 |
| TNN | NM_022093 | 2.552517 |
| TRIOBP | NM_007032 | 2.552229 |
| FAM62B | NM_020728 | 2.551116 |
| ALDH1A3 | NM_000693 | 2.550183 |
| CASP8 | NM_033358 | 2.549711 |
| OLAH | NM_018324 | 2.549638 |
| KIF24 | AK001795 | 2.549379 |
| PARVA | NM_018222 | 2.549024 |
| MTCH2 | NM_014342 | 2.548561 |
| SCCPDH | NM_016002 | 2.546831 |
| CCDC87 | NM_018219 | 2.545636 |
| SLC22A2 | NM_003058 | 2.545021 |
| FAM92A3 | BC059396 | 2.543603 |
| MYRIP | NM_015460 | 2.542672 |
| TMEM51 | NM_018022 | 2.541487 |
| COL11A1 | NM_080629 | 2.540029 |
| WAPAL | NM_015045 | 2.538596 |
| PARD6A | NM_016948 | 2.537382 |
| FLJ20581 | NM_017888 | 2.537328 |
| AMHR2 | NM_020547 | 2.5365 |
| TPCN2 | NM_139075 | 2.536184 |
| PDCD1LG2 | NM_025239 | 2.536077 |
| HILS1 | NM_194072 | 2.535997 |
| IGFBP7 | NM_001553 | 2.535346 |
| KYNU | NM_003937 | 2.535238 |
| CC2D1B | NM_032449 | 2.534258 |
| ENAH | NM_001008493 | 2.534241 |
| ANKRD26 | NM_014915 | 2.533767 |
| CCDC41 | NM_016122 | 2.532868 |
| TMEM80 | NM_174940 | 2.532353 |
| RP11-125A7.3 | NM_001009814 | 2.531612 |
| TRPS1 | NM_014112 | 2.531542 |
| C9orf125 | NM_032342 | 2.531108 |
| PTPLB | NM_198402 | 2.530808 |
| C14orf79 | NM_174891 | 2.528826 |
| SLC22A3 | NM_021977 | 2.528427 |
| DOCK3 | NM_004947 | 2.527917 |
| VNN1 | NM_004666 | 2.527819 |
| RBP7 | NM_052960 | 2.526347 |
| PTPRK | NM_002844 | 2.525842 |
| PRIM1 | NM_000946 | 2.525666 |
| GPATC2 | NM_018040 | 2.52514 |
| FAM44B | NM_138369 | 2.523832 |
| LACTB | NM_171846 | 2.522658 |
| NPEPL1 | NM_024663 | 2.522628 |
| REST | U13877 | 2.522123 |
| C1orf170 | AK123855 | 2.521958 |
| PCSK5 | NM_006200 | 2.52135 |
| KIAA1609 | NM_020947 | 2.521105 |
| MGLL | NM_007283 | 2.521059 |
| EIF2S2 | NM_003908 | 2.520436 |
| RP11-529I10.4 | NM_015448 | 2.520226 |
| DKFZp686D0972 | NM_001017992 | 2.52004 |
| EXOSC2 | NM_014285 | 2.519354 |
| JMY | NM_152405 | 2.519171 |
| KIAA1109 | CR936613 | 2.518385 |
| ZADH2 | NM_175907 | 2.518155 |
| MAPK9 | NM_002752 | 2.516814 |
| STIM2 | NM_020860 | 2.516713 |
| ABLIM2 | NM_032432 | 2.51554 |
| C11orf70 | NM_032930 | 2.515378 |
| C18orf55 | NM_014177 | 2.514839 |
| ZNF354C | NM_014594 | 2.514633 |
| SAA4 | NM_006512 | 2.514354 |
| DENND4C | AK000627 | 2.513603 |
| CAPSL | NM_144647 | 2.512323 |
| TTLL7 | NM_024686 | 2.5123 |
| KIF14 | NM_014875 | 2.512186 |
| SRP72 | NM_006947 | 2.512041 |
| KIAA1841 | BC039298 | 2.512021 |
| FAM101A | NM_181709 | 2.50842 |
| CDCA5 | NM_080668 | 2.507585 |
| PPP2R1B | NM_181699 | 2.507055 |
| KIAA1893 | NM_052899 | 2.507003 |
| FBXL2 | NM_012157 | 2.505583 |
| KLRG1 | NM_005810 | 2.505118 |
| VHL | NM_000551 | 2.503943 |
| MRP63 | NM_024026 | 2.503449 |
| MMACHC | NM_015506 | 2.503248 |
| EVI1 | BX640908 | 2.503143 |
| GLRA3 | NM_006529 | 2.50265 |
| TSEN2 | NM_025265 | 2.501426 |
| ANKRD44 | NM_153697 | 2.500975 |
| FAM43B | NM_207334 | 2.500811 |
| C14orf132 | NM_020215 | 2.500677 |
| RDH5 | NM_002905 | 2.500674 |
| IFT88 | NM_175605 | 2.500523 |
| FAM63B | NM_001040450 | 2.500304 |
| NBR2 | NM_005821 | 2.500135 |
| ST3GAL1 | NM_003033 | 0.398297 |
| LDLR | NM_000527 | 0.398225 |
| CCDC71 | AK023691 | 0.397638 |
| RPL18 | NM_000979 | 0.397607 |
| CYP19A1 | NM_031226 | 0.396143 |
| HOXB2 | NM_002145 | 0.395918 |
| MYBPH | NM_004997 | 0.395548 |
| WDFY4 | BC032420 | 0.395524 |
| HSD11B1 | NM_181755 | 0.395472 |
| ZFPM1 | NM_153813 | 0.394582 |
| BRD2 | NM_005104 | 0.39444 |
| CLK3 | NM_003992 | 0.394051 |
| BCAM | NM_005581 | 0.393723 |
| PEX5L | NM_016559 | 0.393255 |
| FLJ10781 | NM_018215 | 0.392944 |
| TMEM158 | NM_015444 | 0.39288 |
| GIMAP1 | NM_130759 | 0.392823 |
| VEGF | NM_003376 | 0.39224 |
| B4GALT5 | NM_004776 | 0.390809 |
| ZNF408 | NM_024741 | 0.38983 |
| DNM1DN8-2 | AF357221 | 0.389593 |
| DUX4 | NM_033178 | 0.389316 |
| B3GAT3 | NM_012200 | 0.389114 |
| CDK5RAP2 | NM_018249 | 0.388983 |
| HNRPL | NM_001533 | 0.388901 |
| CP | NM_000096 | 0.388587 |
| CD1B | NM_001764 | 0.388254 |
| BBC3 | NM_014417 | 0.387935 |
| C6orf192 | NM_052831 | 0.387564 |
| CRTC1 | NM_025021 | 0.387433 |
| LOC92345 | NM_138386 | 0.386288 |
| PHC2 | NM_198040 | 0.385402 |
| TOM1 | NM_005488 | 0.385086 |
| TLX1 | NM_005521 | 0.38497 |
| CHI3L1 | NM_001276 | 0.384959 |
| PRPF8 | NM_006445 | 0.384597 |
| OTUD7A | NM_130901 | 0.384272 |
| EVL | AL133642 | 0.381504 |
| BMP2 | NM_001200 | 0.380555 |
| ANKRD9 | NM_152326 | 0.380419 |
| CUL7 | NM_014780 | 0.380412 |
| OKL38 | NM_013370 | 0.380189 |
| ADCY9 | NM_001116 | 0.38011 |
| DSC2 | NM_024422 | 0.379915 |
| IRF3 | NM_001571 | 0.379865 |
| ACSM3 | NM_202000 | 0.379831 |
| CAMK1D | NM_020397 | 0.379206 |
| DNHD1 | NM_144666 | 0.377709 |
| SH2D5 | AK124869 | 0.377271 |
| ODF3 | NM_053280 | 0.376912 |
| NOC2L | BC009786 | 0.37466 |
| PDE4B | NM_001037339 | 0.373656 |
| USP49 | NM_018561 | 0.373085 |
| IGF1 | NM_000618 | 0.37286 |
| GCNT3 | NM_004751 | 0.372557 |
| CXCL1 | NM_001511 | 0.372219 |
| IL17C | NM_013278 | 0.371784 |
| C4B | NM_001002029 | 0.371586 |
| GSCL | NM_005315 | 0.370813 |
| C10orf10 | NM_007021 | 0.370232 |
| NMB | NM_021077 | 0.36984 |
| RAVER1 | NM_133452 | 0.367759 |
| PHF15 | NM_015288 | 0.367351 |
| LTBP4 | NM_003573 | 0.367227 |
| PER1 | BC028207 | 0.367176 |
| MGC16275 | BC043607 | 0.366492 |
| IREB2 | BC017880 | 0.366105 |
| STC2 | NM_003714 | 0.365548 |
| LCE5A | NM_178438 | 0.365126 |
| NOL1 | NM_006170 | 0.364004 |
| ARL4C | NM_005737 | 0.362557 |
| IFI44L | NM_006820 | 0.362448 |
| PER2 | NM_003894 | 0.354367 |
| MGC5566 | BC000849 | 0.351501 |
| CD82 | NM_002231 | 0.349913 |
| SERPINB9 | NM_004155 | 0.348721 |
| C11orf66 | NM_145017 | 0.34757 |
| PTPRD | NM_002839 | 0.34587 |
| FLJ37078 | NM_001039212 | 0.345138 |
| GLTSCR2 | NM_015710 | 0.341788 |
| RCOR2 | NM_173587 | 0.341695 |
| SMAD9 | NM_005905 | 0.341333 |
| HCN2 | NM_001194 | 0.340994 |
| CMTM8 | NM_178868 | 0.340617 |
| MGC16384 | BC009492 | 0.340032 |
| MME | NM_007289 | 0.338148 |
| CA11 | NM_001217 | 0.335907 |
| CA9 | NM_001216 | 0.335897 |
| ICAM1 | NM_000201 | 0.334178 |
| PPP1R15A | NM_014330 | 0.331347 |
| CIRBP | AK128423 | 0.329702 |
| PRR5 | NM_015366 | 0.329137 |
| KCNJ8 | NM_004982 | 0.326018 |
| HS2ST1 | NM_012262 | 0.322241 |
| SLC19A3 | NM_025243 | 0.320284 |
| TPD52 | NM_005079 | 0.319042 |
| STAP2 | NM_001013841 | 0.316149 |
| ITGB3 | NM_000212 | 0.311464 |
| C1QTNF4 | NM_031909 | 0.311069 |
| DIO3OS | NR_002770 | 0.31 |
| KCNJ15 | NM_170736 | 0.309356 |
| AKR1C1 | NM_001353 | 0.303981 |
| RGNEF | AB082529 | 0.303769 |
| SLPI | NM_003064 | 0.302915 |
| STARD10 | NM_006645 | 0.30155 |
| PDGFRA | BC015186 | 0.300402 |
| TLR2 | NM_003264 | 0.297949 |
| DUSP4 | NM_001394 | 0.297633 |
| C17orf53 | NM_024032 | 0.295169 |
| GPRC5C | NM_022036 | 0.293371 |
| AMPH | NM_001635 | 0.292131 |
| NDRG2 | NM_201535 | 0.288654 |
| DSU | NM_018000 | 0.287534 |
| DVL3 | NM_004423 | 0.284909 |
| SLC25A37 | AY032628 | 0.283782 |
| CRTAC1 | NM_018058 | 0.282635 |
| EFNA1 | NM_004428 | 0.282522 |
| OSMR | BC010943 | 0.281834 |
| AK128756 | AK128756 | 0.27953 |
| FMO1 | NM_002021 | 0.278017 |
| AQP9 | NM_020980 | 0.276237 |
| PPARGC1A | NM_013261 | 0.275635 |
| SFRP2 | NM_003013 | 0.274026 |
| RNF175 | NM_173662 | 0.269705 |
| GLDN | AK023623 | 0.268654 |
| NEBL | NM_006393 | 0.263543 |
| PIM2 | NM_006875 | 0.260465 |
| PAPPA | NM_002581 | 0.256801 |
| RASD1 | NM_016084 | 0.251117 |
| PLA1A | NM_015900 | 0.238839 |
| CSH1 | NM_022640 | 0.235205 |
| TIFA | NM_052864 | 0.230024 |
| VPS53 | NM_018289 | 0.228571 |
| RNF17 | NM_031277 | 0.223603 |
| EFNA1 | NM_004428 | 0.215829 |
| IL28RA | NM_170743 | 0.206999 |
| RASIP1 | NM_017805 | 0.124212 |
